# A Novel *n*-Hexadecane-Degrading Bacterium Provisionally Named “*Thermalkanevorax longiformis*” Isolated from a Deep-Sea Hydrothermal Vent

**DOI:** 10.3390/microorganisms14081783

**Published:** 2026-08-13

**Authors:** Huilin Wen, Ge Liu, Chaomin Sun, Rui Liu

**Affiliations:** 1 Laboratory of Experimental Marine Biology & Center of Deep Sea Research, Shandong Province Key Laboratory of Marine Biodiversity and Bio-Resource Sustainable Utilization, Institute of Oceanology, Chinese Academy of Sciences, Qingdao 266071, China; 2 Laboratory for Marine Biology and Biotechnology, Qingdao National Laboratory for Marine Science and Technology, Qingdao 266071, China; 3 College of Earth Science, University of Chinese Academy of Sciences, Beijing 100049, China

**Keywords:** *Symbiobacteriaceae*, *n*-hexadecane, degradation, hydrothermal environment

## Abstract

Hydrothermal systems contain diverse alkanes derived from biological and geological processes, providing potential carbon and energy sources for microorganisms. However, cultivated thermophilic alkane-utilizing bacteria from hydrothermal environments remain poorly characterized. In this study, a strain L01 was isolated from a deep-sea hydrothermal vent and identified as an alkane-degrading bacterium. The strain L01 grew at 55 °C and pH 7.0 in medium supplemented with *n*-hexadecane under microoxic conditions. Cells were elongated rods approximately 10–20 μm long and ~0.2 μm wide. Phylogenetic analyses based on 16S rRNA gene sequence similarity (93.11% to the closest described relative), average amino acid identity (AAI, 66.27–66.58%), average nucleotide identity (ANI, 80.80–83.05%), and digital DNA-DNA hybridization (dDDH, 12.5–20.8%) demonstrated that strain L01 represents a previously unrecognized genus within the family *Symbiobacteriaceae*, for which the provisional name “*Thermalkanevorax longiformis*” is proposed. An isotope tracing experiment together with genomics and transcriptomics revealed coordinated physiological and metabolic responses associated with *n*-hexadecane utilization by strain L01. These findings provide a cultivated representative of the family *Symbiobacteriaceae* with physiological and metabolic responses to *n*-hexadecane, thereby providing a basis for further investigation of hydrocarbon metabolism.

## 1. Introduction

Alkanes with carbon chain lengths from C1 to C35 are widely distributed in deep-sea hydrothermal fields along the Atlantic Ocean Ridge, which likely originate from abiotic hydrothermal reactions and the transformation of organic matter under high-temperature conditions [1,2]. These alkanes provide a source of carbon and energy for a variety of bacteria, such as *Pseudomonas aeruginosa*, *Alcanivorax borkumensis*, *Rhodococcus erythropolis*, *Acinetobacter venetianus*, *Desulfatibacillum aliphaticivorans*, *Vibrio* sp., *Psychrobacter* sp., *Dietzia* sp., *Microbacterium* sp., and *Planococcus* sp., many of which are mesophilic [3,4,5,6,7,8,9].

In recent decades, anaerobic alkane-degrading microorganisms have been identified in hydrothermal vents. Rueter et al. (1994) found a thermophilic sulfate-reducing strain TD3, isolated from Guaymas Basin sediment, which can utilize aliphatic and aromatic hydrocarbons [10]. Adams et al. (2013) also found that members of the sulfate-reducing *Deltaproteobacteria* mediate the anaerobic oxidation of short-chain alkanes in MV hydrothermal vent sediments [11]. Schouw et al. (2016) identified an alkane-degrading anaerobic bacterium *Abyssivirga alkaniphila* gen. nov., sp. nov. strain L81 from a deep-sea hydrothermal vent system [12]. Furthermore, in addition to bacteria, anaerobic archaea such as *Ca*. Alkanophaga and *Ca*. Cerberiarchaeum oleivorans have also been found to degrade alkanes at high temperatures [13,14]. Collectively, these findings highlight hydrothermal vents as a reservoir of phylogenetically diverse alkane-degrading microorganisms and provide a foundation for investigating the metabolic potential of additional, as yet uncharacterized microbial taxa.

The family *Symbiobacteriaceae* represents a distinct taxonomic unit within the order *Eubacteriales*, class *Clostridia*, and phylum *Bacillota*. The taxonomic history of this family dates back to 1988, when Suzuki et al. successfully cultivated *Symbiobacterium thermophilum* strain T from compost, marking the first isolation of a thermophilic representative within the genus *Symbiobacterium* [15]. Subsequent studies in 2014 led to multiple novel *Symbiobacterium* strains from shellfish (e.g., *S. ostreiconchae*, *S. turbinis*, and *S. terraclitae*). Finally, the phylogenetic distinctiveness of the genus *Symbiobacterium* culminated in the proposal of the name *Symbiobacteriaceae* fam. nov [16], and only six isolated strains within this family have been successfully obtained. They are characterized by the ability to metabolize various organic compounds; for instance, *S*. *terraclitae*, *S*. *turbinis*, and *S*. *ostreiconchae* utilize yeast extract and peptone, while *S*. *thermophilum* metabolizes glucose, cellobiose, and amino acids [16,17]. Additionally, a representative strain *Caldinitratiruptor microaerophilus* CA62N of the genus *Caldinitratiruptor* can utilize glucose, fructose, mannose, lactate, and pyruvate [18]. However, little is known about the occurrence of *Symbiobacteriaceae* in hydrothermal vents or their potential to utilize alkane.

In this study, a novel thermophilic bacterium, designated strain L01, was isolated from a deep-sea hydrothermal vent and represents a novel genus level within the family *Symbiobacteriaceae*. The morphology of strain L01 was examined by transmission and scanning electron microscopy. The ability of strain L01 to utilize *n*-hexadecane under nearly microoxic conditions was investigated through physiological experiments in combination with genomics, transcriptomics, and metabolomics. This integrated approach was used to characterize its physiological traits and to explore its metabolic potential during alkane utilization. Overall, this study extends the range of source environments and laboratory-observed metabolic traits documented for cultivated members of the family *Symbiobacteriaceae* and provides a new isolate for investigating *n*-hexadecane utilization under controlled conditions.

## 2. Materials and Methods

### 2.1. n-Alkane Analysis of Hydrothermal Vent Sediments

The hydrothermal sediment sample used in this study was collected from the Semyenov-5 hydrothermal area on the Mid-Atlantic Ridge (13.51° N, 44.92° W) at a water depth of approximately 2400 m. Hydrothermal sediment samples were freeze-dried, homogenized, and sieved prior to analysis. Approximately 5 g of sediment was spiked with deuterated *n*-tetracosane as an internal standard and extracted using an Automated Rapid Solvent Extraction System at 120 °C and ~10 MPa with a static extraction time of 10 min and two extraction cycles. The extracts were rotary evaporated to 1 mL and subsequently saponified with 5 mL of a 6% KOH–methanol solution in an 80 °C water bath for 60 min. Subsequently, *n*-alkanes were extracted three times with 12 mL of *n*-hexane, and the combined extracts were concentrated under nitrogen. The extracts were further purified using silica gel column chromatography, eluted with *n*-hexane, dried over anhydrous sodium sulfate, and concentrated to a final volume of 1 mL.

The purified extracts were analyzed using a gas chromatography–mass spectrometry system (GC-MS; QP2010, Shimadzu, Kyoto, Japan) equipped with a DB-5MS fused-silica capillary column (60 m × 0.25 mm × 0.25 μm; Agilent, Santa Clara, CA, USA). The oven temperature program was as follows: 60 °C for 1 min, increased to 200 °C at 7 °C min^−1^, then to 325 °C at 6 °C min^−1^, and then held for 30 min. The injector temperature was maintained at 300 °C, and samples were injected in splitless mode. Mass spectrometric detection was performed in selected ion monitoring (SIM) mode with an ion source temperature of 250 °C, an interface temperature of 300 °C, and a solvent delay of 7.5 min.

### 2.2. Enrichment, Isolation, and Culture of Strain L01

Enrichment cultures were established in 15 mL Hungate tubes by suspending 1 g of hydrothermal vent sediment in sterile seawater and then inoculating 200 μL of the resulting slurry into 10 mL of anaerobic basal medium (BM). The headspace was flushed with a gas mixture of 10% H_2_, 10% CO_2_, and 80% N_2_ to ensure anaerobic conditions. The BM contained (per liter of distilled water) 1.0 g NH_4_Cl, 1.0 g NaHCO_3_, 1.0 g CH_3_COONa, 0.5 g KH_2_PO_4_, 0.2 g MgSO_4_·7H_2_O, 25 g NaCl, 0.1 g CaCl_2_, 0.2 g KCl, 1.7 g NaNO_3_, 1 g yeast extract, 1 g tryptone, and 10 mL modified Wolin’s mineral solution (Table S1) [19]. The pH was adjusted to 7.0 ± 0.2 prior to autoclaving. Cultures were incubated at 55 °C for 15 days.

The enrichment culture was successively transferred seven times by inoculating a 10^−2^ dilution of the preceding culture into fresh medium under the same cultivation conditions. The resulting stable culture was designated strain L01. The purity of strain L01 was subsequently assessed by 16S rRNA gene sequencing, electron microscopic observation, and examination of the whole-genome sequencing data. Specifically, the 16S rRNA gene was independently amplified and sequenced from cultures obtained on three separate occasions. The resulting chromatograms were examined for ambiguous bases and persistent overlapping peaks. In addition, multiple fields were examined by electron microscopy, and the whole-genome sequencing data were evaluated based on the GC-depth and 17-mer frequency distributions.

For routine cultivation, strain L01 was cultivated in basal medium 1 (BM1) supplemented with *n*-hexadecane (>99%) at a final concentration of 0.05% (*v*/*v*). The BM1 contained (per liter of distilled water) 1.0 g NH_4_Cl, 1.25 g Na_2_CO_3_, 1.0 g CH_3_COONa, 0.5 g KH_2_PO_4_, 0.2 g MgSO_4_·7H_2_O, 25 g NaCl, 0.1 g CaCl_2_, 0.2 g KCl, 1.7 g NaNO_3_, 1 g yeast extract, 2 g tryptone, and 10 mL modified Wolin’s mineral solution (Table S1). The pH of the medium was adjusted to 7.0 ± 0.2 prior to autoclaving, and the headspace was flushed with N_2_ to maintain anaerobic conditions.

For the sole-substrate growth experiment, MSM1 mineral medium was used. The MSM1 contained, per liter of distilled water, 1.0 g NH_4_Cl, 0.5 g KH_2_PO_4_, 0.163 g MgCl_2_·6H_2_O, 25.0 g NaCl, 0.1 g CaCl_2_, 0.2 g KCl, 1.25 g Na_2_CO_3_, 1.7 g NaNO_3_, and 10 mL of trace element solution. The pH was adjusted to 7.0. The trace element solution contained, per liter, 0.3 g H_3_BO_3_, 0.59 g MnCl_2_·4H_2_O, 0.15 g CoCl_2_·6H_2_O, 0.09 g ZnCl_2_, 0.01 g CuCl, 0.01 g AlCl_3_, 0.01 g Na_2_MoO_4_·2H_2_O, 0.03 g NiCl_2_·6H_2_O, 0.0002 g Na_2_SeO_3_, and 0.0004 g Na_2_WO_4_·2H_2_O. The solution was adjusted to pH 7.0 and brought to a final volume of 1 L with distilled water. After sterilization and cooling, Kao and Michayluk vitamin solution and *n*-hexadecane were added at final concentrations of 0.1% (*v*/*v*) and 0.05% (*v*/*v*), respectively [20].

### 2.3. Genome Sequencing and Annotation of Strain L01

Cells of strain L01 in the exponential growth phase were harvested by centrifugation at 7000× *g* for 30 min at 4 °C. Cell pellets were submitted to Majorbio Co., Ltd. (Shanghai, China) for genomic DNA extraction and whole-genome sequencing. Genome sequencing was performed using a combination of Illumina short-read and PacBio long-read platforms. De novo genome assembly was conducted using Unicycler (v0.4.8), incorporating PacBio long reads, followed by polishing with Pilon based on Illumina short-read data. Genome circularization was achieved by trimming terminal overlaps, resulting in a finished chromosomal sequence.

Functional annotation was performed by comparing predicted protein sequences against multiple public databases, including NR, Swiss-Prot, EggNOG, KEGG, Pfam, and GO, to assign putative gene functions based on sequence similarity.

### 2.4. 16S rRNA Gene Amplification and Phylogenetic Analysis

The nearly full-length 16S rRNA gene of strain L01 was amplified using the universal bacterial primers 27F (5′-AGAGTTTGATCCTGGCTCAG-3′) and 1492R (5′-GGTTACCTTGTTACGACTT-3′). PCR amplification was performed in a total reaction volume of 20 μL containing 10 μL of Green Taq Mix (Vazyme, Nanjing, China), 2 μL of genomic DNA template, 1 μL of primer 27F (10–20 pmol), 1 μL of primer 1492R (10–20 pmol), and 6 μL of sterile deionized water. The PCR program consisted of an initial denaturation at 95 °C for 10 min followed by 30 cycles of denaturation at 95 °C for 15 s, annealing at 54 °C for 15 s, and extension at 72 °C for 25 s. Amplifications were carried out using a Veriti thermal cycler (Thermo Fisher Scientific, Waltham, MA, USA). PCR products were purified and sequenced by Tsingke Biotechnology Co., Ltd. (Qingdao, China). The obtained 16S rRNA gene sequence was compared with reference sequences available in the NCBI GenBank database using the BLAST (v2.3.0) algorithm.

For phylogenetic analysis, the 16S rRNA gene sequence of strain L01 was aligned with those of closely related taxa within the family *Symbiobacteriaceae* retrieved from the NCBI GenBank database using ClustalW implemented in MEGA X (v10.2.6) [21]. Phylogenetic distances were calculated using the p-distance method, and a neighbor-joining phylogenetic tree was reconstructed. To construct another phylogenetic tree according to 120 conserved proteins of bacteria, a total of 59 genomic sequences including strain L01 and other bacteria in the class *Clostridia* were downloaded from NCBI genome databases and aligned by the GTDB database (release 2.3.2) with automated settings [10.1093/bioinformatics/btac672]. The maximum-likelihood tree was calculated using IQ-TREE (v3.1.3) with the YEAST+F+G4 model and 1000 bootstrap replicates (-bb 1000). All trees were visualized using iTOL (v6) [10.1093/nar/gkae268].

To further assess the phylogenetic relationships between strain L01 and related taxa at the whole-genome level, average amino acid identity (AAI), average nucleotide identity (ANI), digital DNA-DNA hybridization (dDDH), and GTDB-Tk classification analyses were performed between strain L01 and representatives of the closely related genera *Symbiobacterium, Caldinitratiruptor,* and *Thermaerobacter.* The AAI and ANI analyses were conducted using the online tools available on the Majorbio Cloud Platform (https://www.majorbio.com/tools, accessed on 16 April 2024). The dDDH values between strain L01 and its closely related taxonomic neighbors were calculated using the Genome-to-Genome Distance Calculator (GGDC 3.0) online service (https://ggdc.dsmz.de) [22]. The GTDB-Tk was downloaded, installed, and applied according to the instructions on the website (https://github.com/ecogenomics/gtdbtk, GTDB-Tk v2.7.0 with the release 11-RS232, accessed on 5 August 2026).

### 2.5. Morphological Observation

To examine both the overall cellular morphology and the ultrastructural features of strain L01, high-resolution images were obtained using transmission electron microscopy (TEM). For whole-cell observation, 1 mL of cell suspension was harvested by centrifugation at 7000× *g* for 10 min at 25 °C, washed once with sterile water, and centrifuged again under the same conditions. The pellet was resuspended in approximately 50 μL of sterile water, applied onto copper grids for 30 min, air-dried, and then observed directly using a HITACHI TEM system (HT7700, Hitachi, Tokyo, Japan).

For ultrathin sectioning, 200 mL of cell suspension was collected by centrifugation at 7000× *g* for 15 min at 25 °C. The cell pellet was gently fixed in 1 mL of 2.5% glutaraldehyde (Solarbio, Beijing, China) at 4 °C overnight, washed three times with 0.1 M phosphate buffer (15 min each), and postfixed with 1% osmium tetroxide for 1–1.5 h. After three additional washes with 0.1 M phosphate buffer (15 min each), cells were dehydrated sequentially in 50%, 70%, and 90% acetone for 15 min each followed by three changes of 100% acetone for 15–20 min each. Samples were embedded in Epon 812 resin by stepwise infiltration with acetone–resin mixtures (2:1 *v*/*v* for 30 min at 25 °C, 1:2 *v/v* for 1.5 h at 37 °C) followed by pure resin at 37 °C for 2–3 h. Polymerization was carried out sequentially at 37 °C, 45 °C, and 60 °C for 24 h at each temperature. Ultrathin sections (~70 nm) were prepared using a Reichert-Jung Ultracut E ultramicrotome (Vienna, Austria), collected on copper grids, and stained with uranyl acetate for 15 min followed by lead citrate for 15 min prior to observation.

For scanning electron microscopy (SEM), 20 mL of cell suspension was harvested by centrifugation at 7000× *g* for 10 min at 25 °C and washed with 0.1 M phosphate-buffered saline (PBS). The pellet was fixed in precooled 5% glutaraldehyde at 4 °C for 2 h, and then rinsed three times with 0.1 M PBS (10 min each). Samples were dehydrated sequentially in 30%, 50%, 70%, 80%, 90%, and 100% ethanol for 10 min at each step and then subjected to critical-point drying for approximately 3 h. Dried samples were examined using a field-emission scanning electron microscope (Gemini 500, ZEISS, Oberkochen, Germany).

Gram staining was performed using 1 mL of cell suspension harvested by centrifugation at 7000× *g* for 10 min at 25 °C. After removal of most of the supernatant, the concentrated cell suspension was spread onto a clean glass slide, air-dried, and heat-fixed. Smears were stained using a commercial Gram staining kit (Qingdao Hope Bio, Qingdao, China) according to the manufacturer’s instructions and examined under a light microscope (Olympus, Tokyo, Japan).

### 2.6. Growth Under Different Oxygen Conditions

To evaluate the response of strain L01 to different oxygen conditions, three treatments were established using BM1 supplemented with resazurin solution at 500 μL/L and *n*-hexadecane at 0.05% (*v*/*v*). For the oxic treatment, the medium was dispensed into aerobic culture tubes without N_2_ flushing. For the low-oxygen treatment approximating a microoxic environment, the medium was dispensed into sealed Hungate tubes, and the headspace was flushed with N_2_. The anaerobic treatment was prepared similarly, except that a filter-sterilized Na_2_S·9H_2_O stock solution (67 mM) was added at 3% (*v*/*v*), resulting in a final concentration of approximately 2.0 mM. The initial dissolved oxygen concentrations were measured using a Microx 4 trace fiber-optic oxygen meter (PreSens Precision Sensing GmbH, Regensburg, Germany) and were approximately 163.4 ± 4.1, 41.5 ± 2.4, and 0.71 ± 0.0 μmol/L for the oxic, low-oxygen, and anaerobic treatments, respectively. Cultures were incubated at 55 °C for 2 d, and samples were collected at 0, 1, and 2 d. Growth was evaluated by quantifying 16S rRNA gene copy numbers as described in Section 2.7. All treatments were performed with three biological replicates.

### 2.7. qPCR-Based Growth Curve Determination of Strain L01

The growth of strain L01 was estimated by quantitative real-time PCR (qPCR) targeting a specific fragment of the 16S rRNA gene. Genomic analysis revealed that strain L01 contains 9 copies of the 16S rRNA gene per chromosome. Each qPCR was performed in a total volume of 10 μL containing 5 μL SYBR^®^ Green Realtime PCR Master Mix (Toyobo, Osaka, Japan), 3 μL sterile distilled water, 0.5 μL of each primer, and 1 μL of DNA template. Amplification was carried out with an initial denaturation at 95 °C for 1 min followed by 40 cycles of denaturation at 95 °C for 15 s and annealing/extension at 60 °C for 1 min. A ~150 bp fragment of the 16S rRNA gene was amplified using primers 16SF (5′-CATCAGGAAATGGGTGTGG-3′) and 16SR (5′-CCTTTCACCCCTGACTTACC-3′). For standard curve construction, the nearly full-length 16S rRNA gene of strain L01 was cloned into the pUCm-T vector (Sangon Biotech, Shanghai, China) and propagated in *Escherichia coli* DH5α. Recombinant plasmids were purified using a Plasmid Extraction Kit (Tiangen, Beijing, China), and DNA concentrations were determined with a NanoDrop spectrophotometer (IMPLEN, Munich, Germany). Plasmid copy numbers were calculated according to Formula (1) [23]:

(1) Copies μL^−1^ = ((6.02 × 10^23^) × (DNA concentration, ng μL^−1^ × 10^−9^ ))/(DNA length(bp) × 660)

The recombinant plasmid was serially diluted 10-fold to generate eight concentration gradients. Standard curves were constructed by plotting Ct values against the corresponding plasmid copy numbers and were used for absolute quantification (Figure S3). The recombinant plasmid copy number was assumed to be equivalent to the 16S rRNA gene copy number (Supplementary Dataset).

For growth curve determination, 1 mL of bacterial culture was harvested by centrifugation at 7000× *g* for 30 min at 25 °C. The total genomic DNA was extracted from the cell pellet using the boiling method and resuspended in 50 μL of sterile distilled water. The extracted DNA was used as the template for qPCR analysis to determine the 16S rRNA gene copy number.

Strain L01 was cultivated in MSM1 mineral medium supplemented with Kao and Michayluk vitamin solution at 0.1% (*v*/*v*). Experimental cultures received 0.05% (*v*/*v*) *n*-hexadecane, whereas the no-hexadecane controls received the same volume of medium without *n*-hexadecane. Both treatments contained identical concentrations of vitamins, mineral salts, and nitrate and differed only in the addition of *n*-hexadecane. Cultures were incubated in sealed bottles at 55 °C, and the growth of the strain L01 was quantified by qPCR targeting the 16S rRNA gene at days 0, 1, 3, 6, 7, 9, and 10.

### 2.8. Determination of n-Hexadecane Degradation

For the degradation assay, 5 μL of *n*-hexadecane was added to 10 mL of BM1. Abiotic controls were prepared without inoculation, while experimental cultures were inoculated with strain L01 at 4% (*v*/*v*). After incubation at 55 °C, residual *n*-hexadecane was extracted by adding 2 mL of *n*-hexane to each culture and allowing the mixture to extract overnight. The upper organic phase was collected for analysis. Quantification of *n*-hexadecane was performed using an Agilent 8860 gas chromatograph equipped with a flame ionization detector (FID) and an HP-5 capillary column (30 m × 320 μm × 0.25 μm; Agilent Technologies, Santa Clara, CA, USA). Analytical-grade *n*-hexadecane (≥99.5%, Macklin, Shanghai, China) dissolved in chromatographic-grade *n*-hexane (≥99%, Macklin, China) was used to prepare standard solutions. Nitrogen was used as the carrier gas. Samples were injected in split mode (split ratio 10:1) at an inlet temperature of 200 °C with a split flow rate of 65 mL/min. The oven temperature program was as follows: 70 °C for 0.5 min, ramped to 190 °C at 20 °C/min, and then held for 6.25 min. The FID temperature was set at 250 °C, and the injection volume was 1 μL. A calibration curve was generated by analyzing serially diluted *n*-hexadecane standards (0–11,997.77 μmol/L) under identical chromatographic conditions, and linear regression was performed using peak area versus concentration (Figure S4). Quantification of extracted samples was conducted using the external standard method. At least two chromatograms of the analyzed samples were recorded.

The *n*-hexadecane degradation rate was calculated using Equation (2) [24]:

(2) Degradation rate (%) = (C_0_ − C_t_)/C_0_ × 100

where $C_{0}$ represents the *n*-hexadecane concentration in abiotic controls, and $C_{t}$ represents the residual *n*-hexadecane concentration in inoculated cultures at time t.

### 2.9. Identification of Marker Genes Involved in Hydrocarbon Degradation

Putative marker genes involved in hydrocarbon degradation were searched in the genome of strain L01 using HMMER (v3.3.1) against the CANT-HYD database [25]. Hits with a full-sequence E-value < 1 × 10^−10^ were retained as candidate homologs for subsequent analyses.

Conserved sequence motifs were analyzed by comparing the reference AhyA sequence from *Desulfococcus oleovorans* Hxd3 (WP_012173623.1) in the CANT-HYD database with the candidate homologous protein sequences from strain L01 using the MEME Suite (version 5.5.9) [26].

### 2.10. Transcriptome Sequencing and Differential Expression Analysis

Strain L01 was cultivated in BM1 supplemented with a trace amount of *n*-hexadecane (15 μL per 200 mL medium) for 24 h. Subsequently, three parallel inoculated cultures in the experimental group were further supplemented with an additional 85 μL of *n*-hexadecane, and three parallel control cultures were not amended with additional *n*-hexadecane. Cultures from both groups were harvested at 1 h and 4 h after *n*-hexadecane induction. Cells were collected by centrifugation at 7000× *g* for 30 min at 4 °C, and the pellets were submitted to Majorbio Co., Ltd. (Shanghai, China) for total RNA extraction and transcriptome sequencing.

Functional annotation of expressed genes was performed by comparison against multiple databases, including NR, Swiss-Prot, EggNOG, KEGG, Pfam, and GO databases. Differential gene expression analysis between experimental and control groups was conducted using DESeq2. All transcriptomic experiments were performed with three biological replicates.

### 2.11. RT-qPCR Analysis of Selected Genes

Strain L01 was first cultivated in BM1 supplemented with a low amount of *n*-hexadecane (15 μL per 200 mL of medium) for 24 h. The cultures were then divided into experimental and control groups, with three independent biological replicates per group. An additional 85 μL of *n*-hexadecane was added to each experimental culture, whereas no additional *n*-hexadecane was added to the control cultures. Cells from both groups were harvested 24 h after the additional *n*-hexadecane amendment by centrifugation at 7000× *g* for 30 min at 4 °C.

Total RNA was extracted using TRIzol reagent. Briefly, the cell pellet was resuspended in 1 mL of TRIzol reagent (HL-BIOTECH, Qingdao, China) and then 0.2 mL of chloroform was added. After thorough mixing and phase separation by centrifugation, the upper aqueous phase was transferred to a new RNase-free tube. RNA was precipitated by adding an equal volume of isopropanol, washed with 75% ethanol, and subsequently dissolved in RNase-free water.

The extracted RNA was reverse-transcribed into cDNA using HiScript II Q RT SuperMix for qPCR (Vazyme, Nanjing, China) according to the manufacturer’s instructions. The resulting cDNA was used as the template for quantitative real-time PCR. The reaction mixture and amplification conditions were the same as those described in Section 2.7, except that cDNA was used as the template. The 16S rRNA gene was used as the internal reference gene. Relative expression levels of the selected genes were calculated using the 2^−ΔΔCt^ method [27], with the cultures without additional *n*-hexadecane serving as the calibrator group. Three biological replicates were analyzed for each treatment.

### 2.12. Metabolomic Analysis Following n-Hexadecane Addition

Both control and experimental groups were cultured in BM1 supplemented with 1 g/L succinate for 2 days. Subsequently, 100 μL of *n*-hexadecane was added to 200 mL of culture in the experimental group, whereas no substrate was added to the control group. After an additional 6 h of incubation, cells were harvested by centrifugation at 7000× *g* for 30 min at 4 °C. The supernatant was discarded, and cell pellets were washed twice with 0.1 M PBS and centrifuged at 7000× *g* for 5 min at 4 °C. The resulting pellets were submitted to Novogene Co., Ltd. (Beijing, China) for untargeted metabolomic analysis.

Metabolites were extracted by resuspending the cell pellets in 300 μL of 80% (*v*/*v*) methanol. Samples were flash-frozen in liquid nitrogen for 5 min, thawed on ice, vortexed for 30 s, and ultrasonicated for 6 min. After centrifugation at 3000× *g* for 1 min at 4 °C, the supernatants were collected and lyophilized. The dried extracts were reconstituted in 70 μL 10% (*v*/*v*) methanol and subjected to LC-MS analysis [28,29].

UHPLC-MS/MS analyses were performed using a Vanquish UHPLC system (Thermo Fisher Scientific, Waltham, MA, USA) coupled to an Orbitrap Q Exactive™ HF-X mass spectrometer (Thermo Fisher Scientific, Waltham, MA, USA). Chromatographic separation was achieved on a Hypersil Gold column (100 × 2.1 mm, 1.9 μm) at a flow rate of 0.2 mL/min using a 12 min linear gradient. The mobile phases consisted of eluent A (0.1% formic acid in water) and eluent B (methanol) for both positive and negative ion modes. The gradient program was as follows: 2% B for 1.5 min, 2% to 85% B for 3 min, 85% to 100% B for 10 min, 100% to 2% B at 10.1 min, and 2% B at 12 min. The mass spectrometer was operated in both positive and negative ion modes with a spray voltage of 3.5 kV, capillary temperature of 320 °C, sheath gas flow rate of 35 psi, auxiliary gas flow rate of 10 L/min, S-lens RF level of 60, and auxiliary gas heater temperature of 350 °C.

Raw UHPLC-MS/MS data were processed using XCMS for peak detection, retention time alignment, and quantification. Metabolites were identified by comparison with a self-built high-quality MS/MS spectral database (NovoMetDB) based on adduct ions and a mass tolerance of 10 ppm. Background ions were removed using blank samples. Quantitative data were normalized as follows: Relative peak areas = Raw quantitative value of samples/(The sum of quantitative value of samples/The sum of quantitative value of QC). Metabolites with coefficients of variation (CVs) greater than 30% in quality control (QC) samples were excluded. The remaining metabolites were retained for downstream analysis and annotated using the KEGG, HMDB, and LIPID MAPS databases.

### 2.13. Metabolomic Analysis of ^13^C-Labeled n-Hexadecane Assimilation

Strain L01 was incubated with 3367 μmol/L hexadecane-1,2-^13^C_2_ (Sigma-Aldrich, St. Louis, MO, USA) or unlabeled *n*-hexadecane for 9 days. Bacterial pellets (approximately 10^10^ cells) were collected by centrifugation (7000× *g*, 10 min, 4 °C), flash-frozen in liquid nitrogen, and subjected to metabolomic analysis by Shanghai Biotree Biotech (Shanghai, China).

For metabolite extraction, approximately 10^10^ cells were resuspended in 1000 μL extraction solvent (MeOH/ACN/H_2_O, 2:2:1, *v*/*v*/*v*) containing deuterated internal standards. Samples were homogenized with beads (4 min, 35 Hz) and subjected to repeated freeze–thaw cycles followed by sonication and protein precipitation at −40 °C. After centrifugation (13,800× *g*, 15 min, 4 °C), the supernatant was collected for LC-MS/MS analysis. Quality control (QC) samples were prepared by mixing equal aliquots of all extracts.

Polar metabolites were analyzed using a UHPLC system (Vanquish, Thermo Fisher Scientific, Waltham, MA, USA) coupled to an Orbitrap Exploris 120 mass spectrometer. Chromatographic separation was performed on a Waters ACQUITY UPLC BEH Amide column (2.1 × 50 mm, 1.7 μm) with a binary solvent system consisting of 25 mmol/L ammonium acetate with ammonia hydroxide (pH 9.75) (A) and acetonitrile (B). Data were acquired in both positive and negative electrospray ionization modes using information-dependent acquisition (IDA) with a full MS resolution of 60,000 and an MS/MS resolution of 15,000.

Raw data were converted to mzXML format using ProteoWizard and processed using an in-house R-based pipeline (XCMS framework) for peak detection, alignment, and quantification [30]. Metabolite annotation was performed using the BiotreeDB (v4.0) database. The detected ^13^C-labeled metabolites were selected and classified based on annotations from KEGG, LIPID MAPS, HMDB, and EMBL-EBI ChEBI databases.

### 2.14. Determination of Nitrate and Sulfate Concentrations

To determine changes in nitrate and sulfate concentrations during cultivation of strain L01, inoculated or uninoculated BM1 supplemented with *n*-hexadecane (0.05%, *v*/*v*) was determined using the Ion Chromatography system (ICS5000+, Thermo Fisher Scientific, Waltham, MA, USA) equipped with an integrated capillary pump and a suppressed conductivity detector. Analyses were performed using a Dionex IonPac AS11-HC RFIC analytical column (4 × 250 mm) protected by a Dionex IonPac AG11-HC RFIC guard column (4 × 50 mm). Separation was achieved using NaOH (100 mM) rinsing solution at a constant flow rate of 1.0 mL/min, and the injection volume was 25 μL. To reduce background noise and enhance sensitivity, a continuous self-regenerating suppressor was employed in the recycle mode.

For temporal analysis, 1 mL of culture supernatant was collected initially and after 10 days incubation, with three biological replicates for each time point. Prior to analysis, the culture supernatant was filtered through a 0.22 μm polyethersulfone membrane to remove cells and particulate matter. Nitrate or sulfate peaks were identified by retention time comparison with certified standards and quantified via external calibration curves, respectively. Net consumption was calculated as shown in Equation (3):

(3) Net consumption (%) = (C_0_ − C_10_)/C_0_ × 100

where C_0_ and C_10_ represent the concentrations at the beginning of incubation and after 10 days, respectively.

### 2.15. Statistical Analysis

Statistical analysis was performed using OriginPro 2026 software (OriginLab Corporation, Northampton, MA, USA). Unless otherwise indicated, all experiments were conducted using three independent biological replicates (n = 3), and data are presented as the mean ± standard deviation. The normality was assessed using the Shapiro–Wilk test before statistical analyses. For experiments involving treatment and incubation time as two independent factors, differences were evaluated using one-way analysis of variance (ANOVA) followed by Tukey’s multiple-comparison test. Exact *p*-values were designated at *p* < 0.05 (*), *p* < 0.01 (**), or *p* < 0.001 (***).

## 3. Results

### 3.1. Isolation of Strain L01

A sediment sample collected from the hydrothermal field along the Mid-Atlantic Ridge was suspended in sterile seawater and inoculated into 10 mL of basal medium (BM). Following seven successive transfers using a 10^−2^ dilution at each transfer, a stable culture of designated strain L01 was obtained. Repeated 16S rRNA gene sequencing yielded concordant sequences with well-resolved chromatographic peaks and no overlapping signals. Electron microscopic observations revealed a morphologically homogeneous population, with no distinct additional cell morphotype observed. The single concentrated GC-depth distribution and a single major 17-mer peak also showed no obvious secondary genomic population. Collectively, these observations support the purity of strain L01.

### 3.2. Phylogenetic and Genomic Placement of Strain L01 Within the Family Symbiobacteriaceae

**Figure 1:**
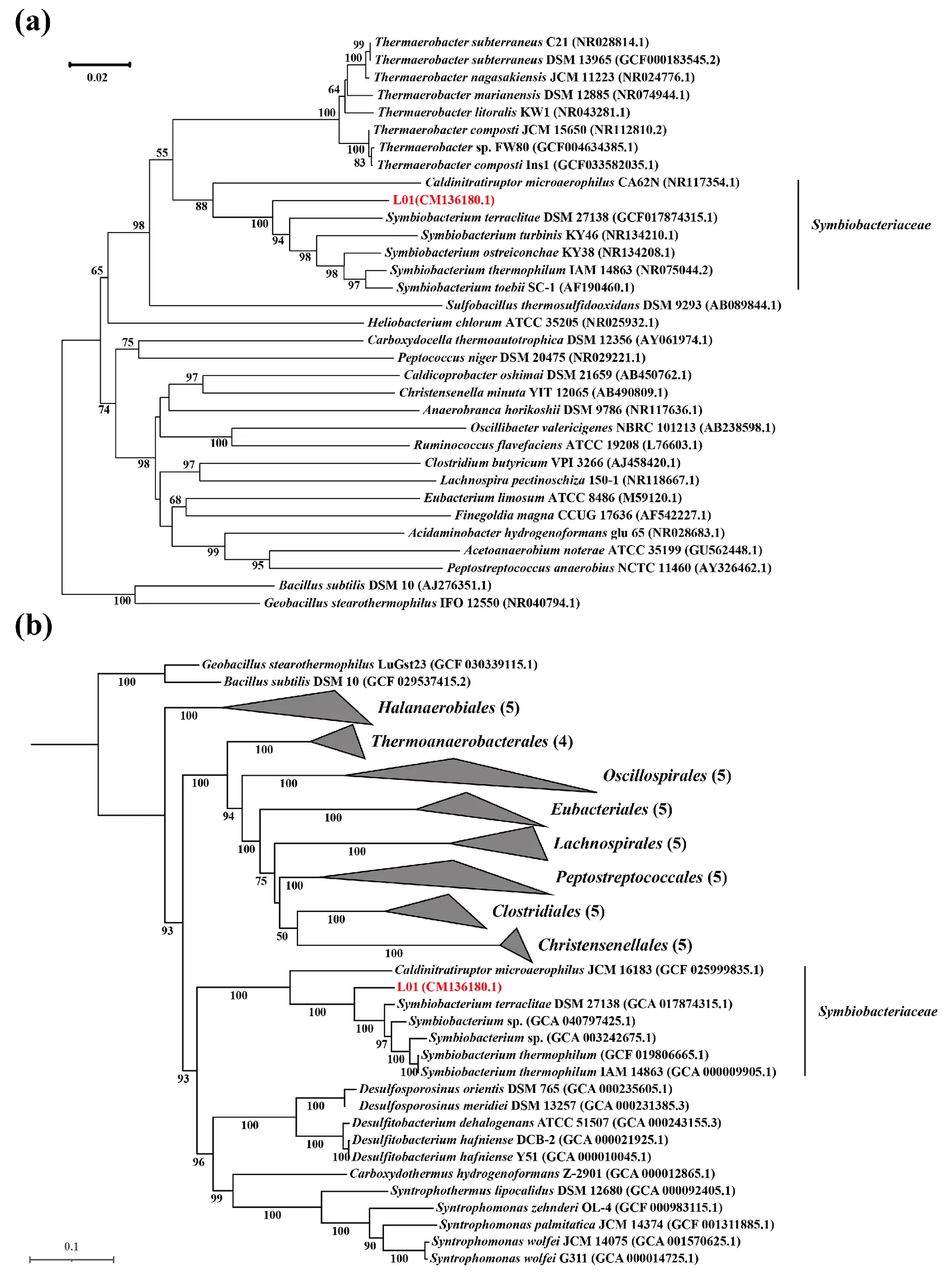
(**a**) Neighbor-joining phylogenetic tree based on 16S rRNA sequences showing the phylogenetic position of strain L01 among members of the family *Symbiobacteriaceae* and related taxa. The 16S rRNA gene sequence of strain L01 used is included in its complete genome sequence deposited in GenBank under accession number CM136180.1. Bootstrap values (≥50%) based on 1000 replicates are indicated at branch nodes. *Bacillus subtilis* DSM 10 and *Geobacillus stearothermophilus* IFO 12550 are used as outgroup taxa. (**b**) Maximum-likelihood phylogenomic tree based on the concatenated amino acid sequences of 120 conserved bacterial proteins showing the phylogenetic position of strain L01 relative to members of the family *Symbiobacteriaceae*. The tree was reconstructed using IQ-TREE under the YEAST+F+R4 substitution model. Bootstrap values (≥50%) based on 1000 ultrafast bootstrap replicates are indicated numerically at branch nodes. Selected clades are collapsed for clarity, and numbers in parentheses indicate the numbers of genomes represented by the corresponding collapsed clades. The strains included in the collapsed clades are listed in the Supplementary Dataset. The family *Symbiobacteriaceae* is indicated in the tree. The scale bar in (**a**) represents 0.02 nucleotide differences per site, and the scale bar in (**b**) represents 0.1 amino acid substitutions per site.

**Figure 2:**
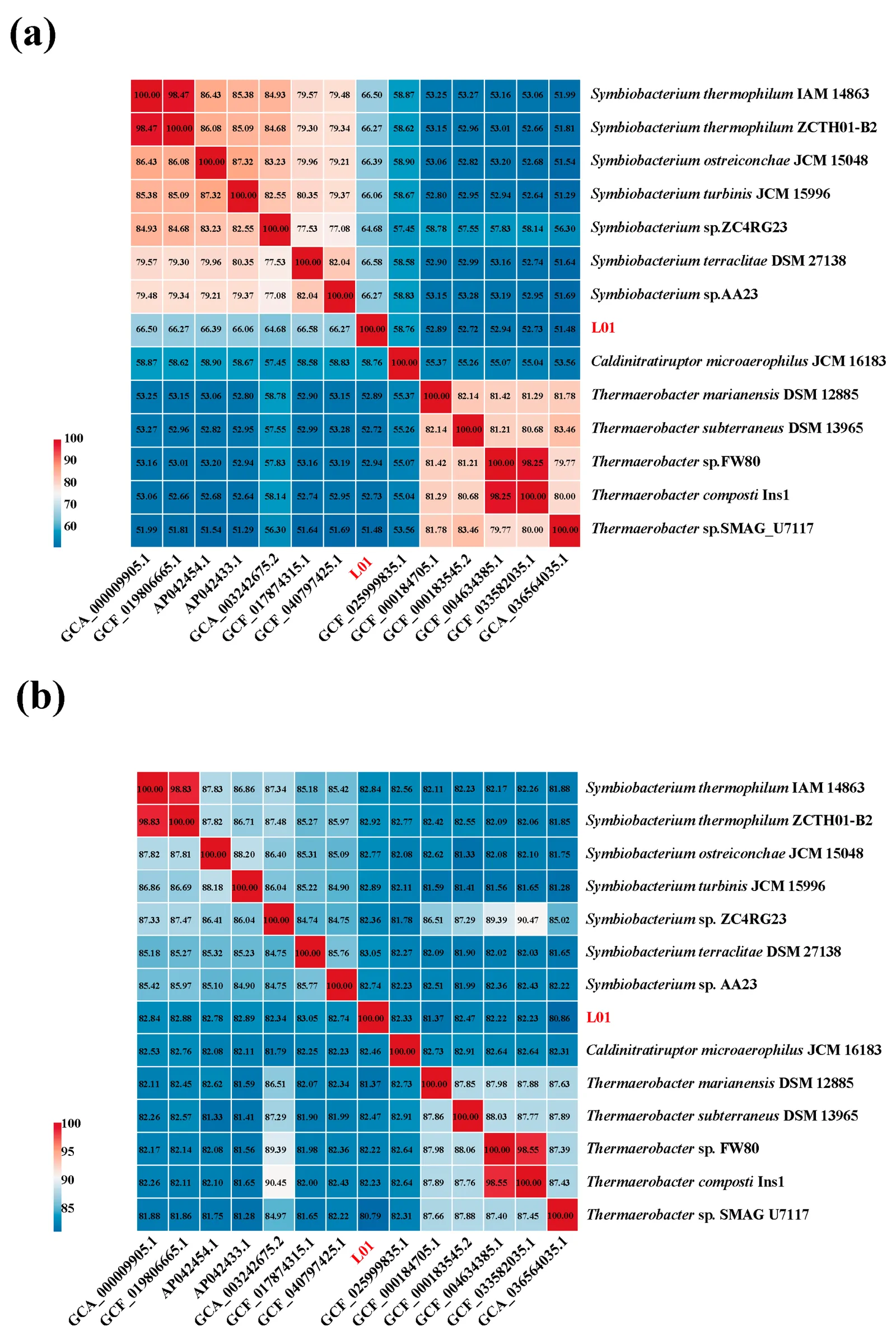
(**a**) Average amino acid identity (AAI) analysis between strain L01 and representative strains of the genus *Symbiobacterium*, *Caldinitratiruptor*, and *Thermaerobacter*. The AAI values are shown in each cell. A color gradient is used to visualize genomic similarity, where deeper red indicates higher AAI values and thus greater genomic relatedness, while lighter colors represent lower similarity. The clear separation of L01 from the described *Symbiobacterium* species and closely related genera at the AAI level supports its placement as a deeply divergent lineage at the genus level. (**b**) Average nucleotide identity (ANI) analysis between strain L01 and closely related strains. The ANI values are shown in each cell. A color gradient is used to visualize genomic similarity, where deeper red indicates higher ANI values and thus greater genomic relatedness, while lighter colors represent lower similarity.

Analysis of the 16S rRNA sequence (1444 bp) of strain L01 revealed that it belongs to the family *Symbiobacteriaceae* and shares 93.11% sequence identity (the highest in the NR database of the NCBI) with *Symbiobacterium ostreiconchae* JCM 15048 (AP042454.1). Phylogenetic analysis was conducted based on 16S rRNA of strain L01 and other species in the family *Symbiobacteriaceae*. Because the family currently comprises limited species, several representatives from other families were also included. Although strain L01 clustered as a sister clade with members of the genus *Symbiobacterium*, it formed an independent branch within this lineage and was clearly separated from the genus *Caldinitratiruptor* (Figure 1a). To further resolve the phylogenetic position of strain L01, phylogenomic analysis was performed with expanded taxon sampling comprising 59 genomes, including representatives of *Symbiobacteriaceae*, additional families within *Eubacteriales*, and several other orders within the class *Clostridia* (Figure 1b). The maximum-likelihood tree based on the concatenated amino acid sequences of 120 conserved bacterial proteins placed strain L01 as a distinct lineage sister to the *Symbiobacterium* clade with 100% ultrafast bootstrap support. This topology was consistent with the 16S rRNA gene phylogeny and supported the phylogenetic distinction of strain L01 from the currently included representatives of *Symbiobacterium*. To further evaluate its genomic relatedness, AAI values were calculated between the genome of strain L01 and representative genomes in the *Symbiobacteriaceae* family. Strain L01 shared only 66.27–66.58% identities with recognized *Symbiobacterium* species (Figure 2a), well below the threshold typically associated with species-level delineation and near the lower boundary proposed for genus-level affiliation [31]. Moreover, its AAI values (approximately 53.56–55.37%) with neighboring genera (Figure 2a), including *Caldinitratiruptor* and *Thermaerobacter*, were even lower than genus boundaries defined within the 60–80% AAI range [32]. Additionally, ANI and dDDH analyses were also performed for polyphasic taxonomic analysis. The ANI value observed between strain L01 and reference strains was 80.8–83.05% (Figure 2b), and the corresponding dDDH value was 12.5–20.8% (Table S2). Both metrics fell substantially below the established species boundaries of 95–96% for ANI and 70% for dDDH [33,34]. Meanwhile, the taxonomy of strain L01 was only classified at the family level after analysis by GTDB-Tk (Supplementary Dataset). Taken together, these phylogenetic and genomic analyses support the placement of strain L01 in a previously unrecognized genus within the family *Symbiobacteriaceae*. The provisional name “*Thermalkanevorax longiformis*” is proposed for strain L01. The strain has been deposited in the Marine Culture Collection of China under accession number MCCC 1K10379.

### 3.3. Morphological and Physiological Characteristics of Strain L01

**Figure 3:**
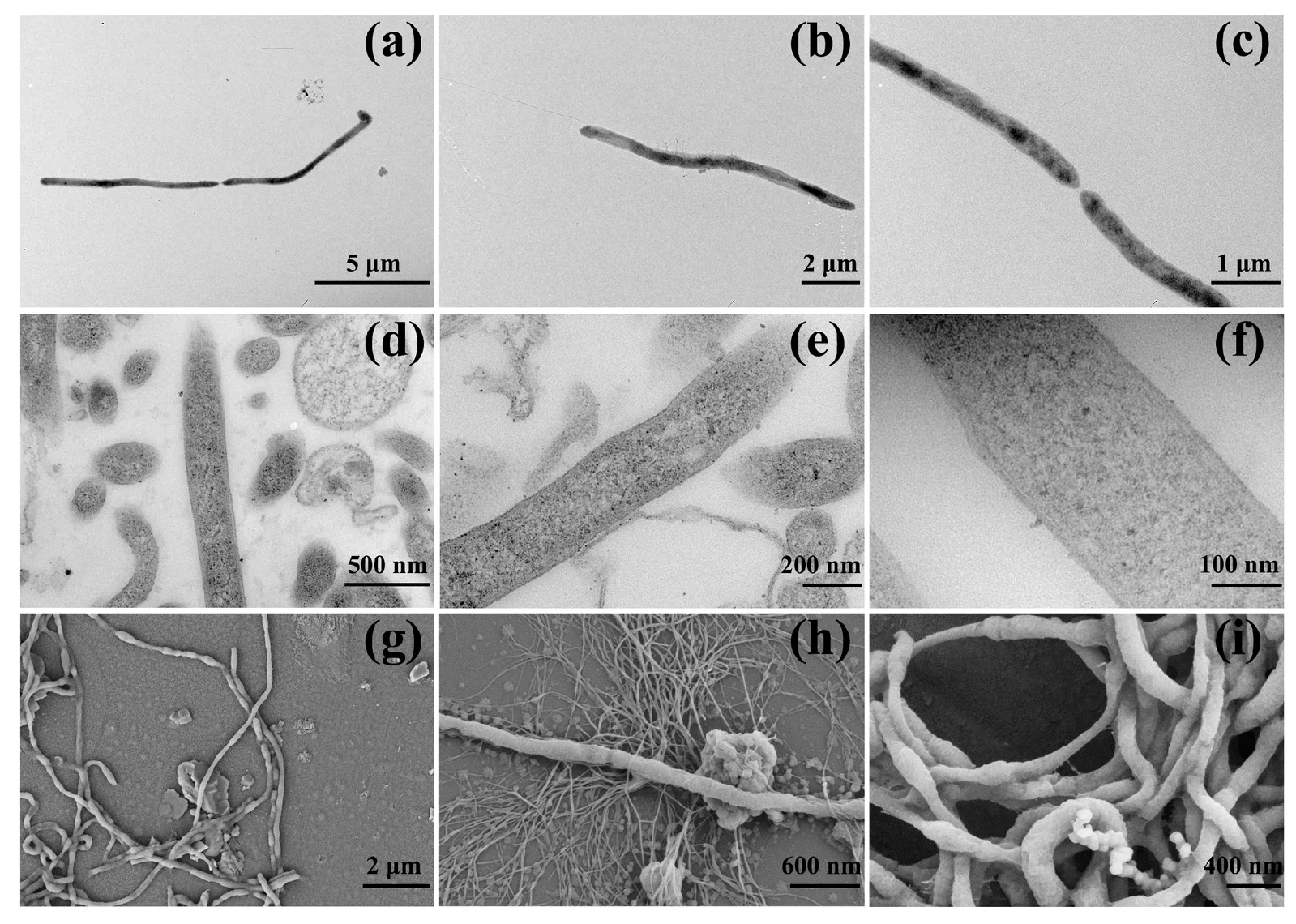
Microscopic observations of strain L01 obtained using transmission electron microscopy (TEM) and scanning electron microscopy (SEM). (**a**–**c**) TEM images showing that cells of strain L01 are slender rods with a single terminal flagellum. Frequently, close cell-to-cell contact or physical connections between neighboring cells were observed. (**d**–**f**) Ultrathin-section TEM images of strain L01. (**g**–**i**) SEM images showing that cells form intertwined filamentous networks with extensive physical contacts. Filamentous or flagellum-like structures were distributed on the cell surface and within cell aggregates.

Morphological characterization via TEM revealed that cells of strain L01 are slender, elongated rods approximately 10–20 μm in length and ~0.2 μm in diameter (Figure 3b,d). Occasional observation of a single terminal flagellum suggests that L01 is motile, a trait further supported by the SEM, which revealed numerous flagellum-like appendages distributed around both individual cells and aggregates (Figure 3g–i). Genome analysis further supported the presence of flagella and motility, with a total of 102 genes identified as being involved in flagellar biosynthesis and chemotaxis (Supplementary Dataset). Both TEM and SEM analyses consistently showed frequent associations with neighboring cells, suggesting a strong tendency for aggregation and potential cooperative behavior (Figure 3a,c,g). Ultrathin-section TEM further revealed electron-dense intracellular structures and intimate connections between adjacent cells (Figure 3d–f). Interestingly, upon entering the stationary growth phase, strain L01 occasionally formed necklace-like filamentous chains composed of serially aligned spherical bodies (Figure S1). This feature resembles the endospore-like cellular structure reported in *S*. *thermophilum*, a member of the same family [17]. Gram staining showed that strain L01 was Gram-positive (Figure S2).

**Figure 4:**
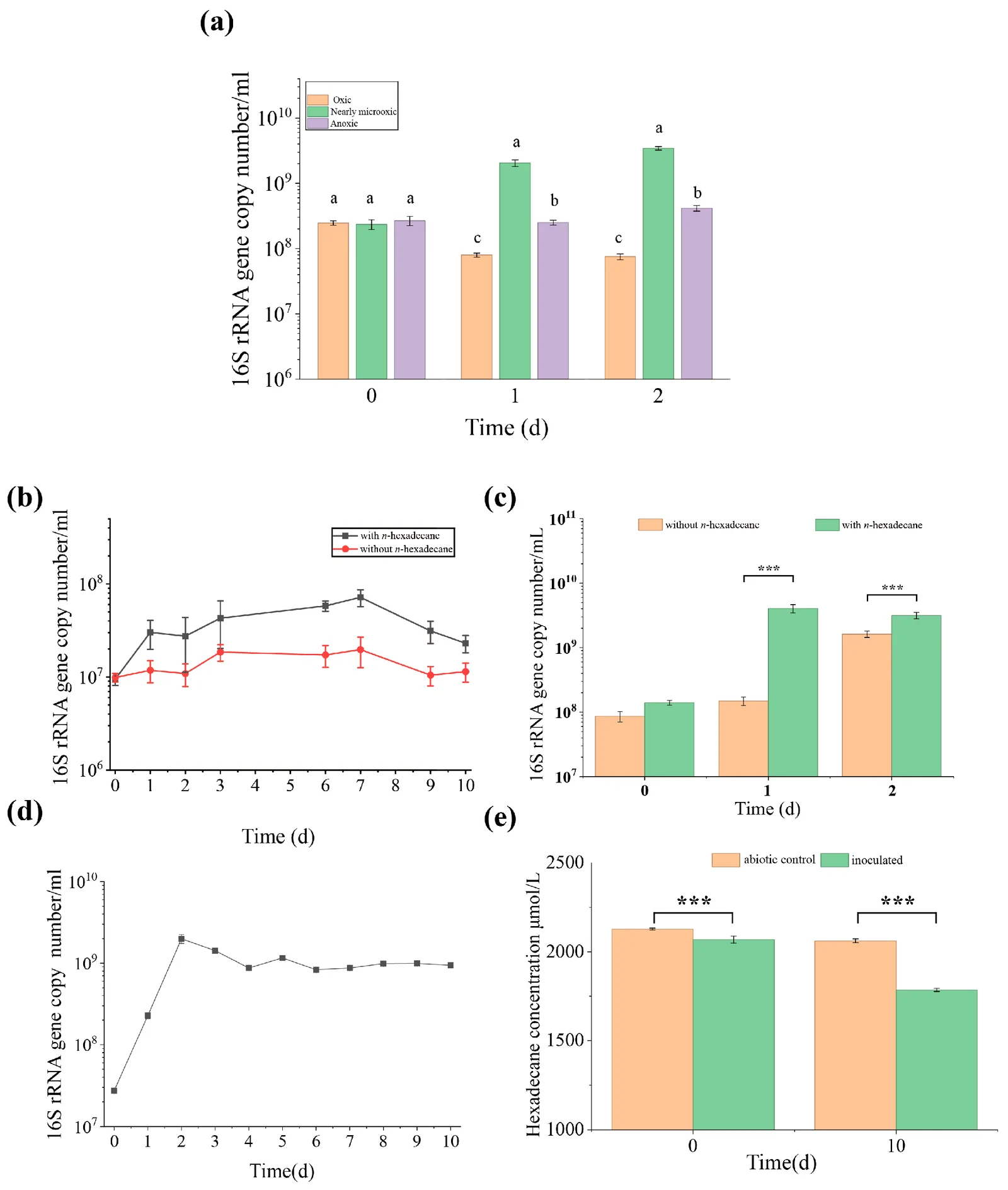
Growth of strain L01 and residual *n*-hexadecane during incubation. (**a**) Growth of strain L01 under different oxygen conditions. Data are presented as mean ± SD (n = 3). Different lowercase letters indicate significant differences among oxygen conditions at the same time point (one-way ANOVA with Tukey’s test, *p*-value < 0.001). (**b**) Growth curve of strain L01 cultivated in MSM1 medium supplemented with or without hexadecane. (**c**) Growth of strain L01 in BM1 supplemented with or without *n*-hexadecane, determined by 16S rRNA gene copy numbers at 0, 1, and 2 d ( *p* < 0.001 (***)). (**d**) Growth curve of strain L01 cultivated in hexadecane-supplemented BM1 by strain L01 during cultivation. Data are presented as mean ± standard deviation. (**e**) Residual *n*-hexadecane during strain L01 incubation (*p* < 0.001 (***)).

Growth of strain L01 was compared under oxic, microoxic, and anoxic conditions, with measured dissolved oxygen concentrations of 163, 41, and 0.71 μmol L^−1^, respectively. The highest growth was observed under the nearly microoxic condition, suggesting that strain L01 preferentially grows under near-microoxic conditions (Figure 4a).

A comprehensive comparison of the phenotypic and physiological characteristics of strain L01 with related members of the family *Symbiobacteriaceae* is presented in Table S3 [16,18,35]. Strain L01 was distinguished from all currently described relatives by a combination of characteristics: It was the only Gram-positive strain among the compared taxa, whereas all other members were reported as Gram-negative. Strain L01 was able to utilize *n*-hexadecane, a phenotype not reported for other members of the family. The strain grew optimally at 55 °C under nearly microoxic conditions. Its optimal growth temperature was similar to those of *S*. *ostreiconchae* and *S*. *terraclitae*, whereas its microaerophilic growth was different from that of most members of the genus *Symbiobacterium*.

### 3.4. Growth Supported by and Consumption of n-Hexadecane by Strain L01

GC-MS analysis of the source sediment detected *n*-alkanes ranging from C9 to C32, with a total concentration of 4403.7 ng/g (Table S4). The quantified C9–C17 fraction totaled 1286.1 ng/g and was dominated by *n*-hexadecane (449.8 ng/g), *n*-decane (340.6 ng/g), and *n*-undecane (138.6 ng/g). Other C9–C17 *n*-alkanes were detected at concentrations ranging from 35.5 to 85.8 ng/g. *n*-Hexadecane accounted for approximately 35.0% of the C9–C17 fraction and 10.2% of the total C9–C32 *n*-alkanes. Given the occurrence of *n*-hexadecane in the source sediment, its effect on the growth of strain L01 was subsequently examined.

To determine whether *n*-hexadecane could support the growth of strain L01 in the absence of auxiliary organic substrates, strain L01 was cultivated in MSM1 mineral medium without yeast extract, tryptone, or acetate. Both the *n*-hexadecane treatment and the no-hexadecane control received the same concentration of Kao and Michayluk vitamin solution. In the presence of *n*-hexadecane, the 16S rRNA gene copy number increased from 9.48 × 10^6^ copies mL^−1^ at day 0 to a maximum of 7.18 × 10^7^ copies mL^−1^ at day 7, corresponding to an approximately 7.6-fold increase (Figure 4b). In contrast, the no-hexadecane control increased from 9.87 × 10^6^ copies mL^−1^ to 1.97 × 10^7^ copies mL^−1^ at day 7. At this time point, cell abundance in the *n*-hexadecane treatment was approximately 3.8-fold higher than that in the control. These results indicate that *n*-hexadecane supported slow but measurable growth of strain L01 under defined mineral conditions. In nutrient-containing BM1, the addition of *n*-hexadecane further accelerated the growth of strain L01 compared with cultures without *n*-hexadecane (Figure 4c).

To further determine whether strain L01 can degrade *n*-hexadecane, degradation experiments were conducted in BM1 supplemented with 0.05% *n*-hexadecane (*v*/*v*), and growth was monitored over time. The copy number of 16S rRNA gene increased from approximately 2.7 × 10^7^ at 0 d to 2.0 × 10^9^ at 2 d and then slightly declined at later time points, although it remained markedly higher than the initial inoculation level (Figure 4d). Residual *n*-hexadecane was quantified by gas chromatography. Based on the *n*-hexadecane concentration in abiotic controls, the degradation rate was 10.4% at 10 d (Figure 4e), corresponding to the consumption of approximately 216.9 μmol/L *n*-hexadecane.

### 3.5. Genomic Potential and Transcriptomic Analysis for Alkane Degradation

Strain L01 harbors a single circular chromosome of 4.3 Mbp with a GC content of 65.75%, and no plasmid was detected. The circular genome map is shown in Figure S5. The genome annotation of strain L01 was additionally examined for known oxygen-dependent enzymes associated with aerobic *n*-alkane oxidation. No genes were annotated as alkane hydroxylases (AlkB or CYP153), long-chain alkane hydroxylases (LadA), or flavin-binding monooxygenases (AlmA). Likewise, genes associated with the canonical anaerobic activation of long-chain alkanes were not detected [36] (pp. 782–793), [37]. In contrast, eight genes were identified to encode a putative alkane C2-methylene hydroxylase (AhyA) by using HMMER search against the CANT-HYD database (Table S4) [25]. The proteins encoded by these genes were found to share two conserved motifs with the reference AhyA protein from *D*. *oleovorans* Hxd3 (*Do*AhyA) (Figure S6). In addition, two genes encoding an alcohol dehydrogenase (gene 2027) and an aldehyde dehydrogenase (gene 3698) were also identified in the genome of strain L01. Moreover, the strain L01 genome harbors genes encoding enzymes for fatty acid β-oxidation, the tricarboxylic acid (TCA) cycle, and the reductive TCA (rTCA) cycle (Supplementary Dataset). Collectively, these genomic features identified several candidate genes potentially associated with alkane metabolism in strain L01.

**Figure 5:**
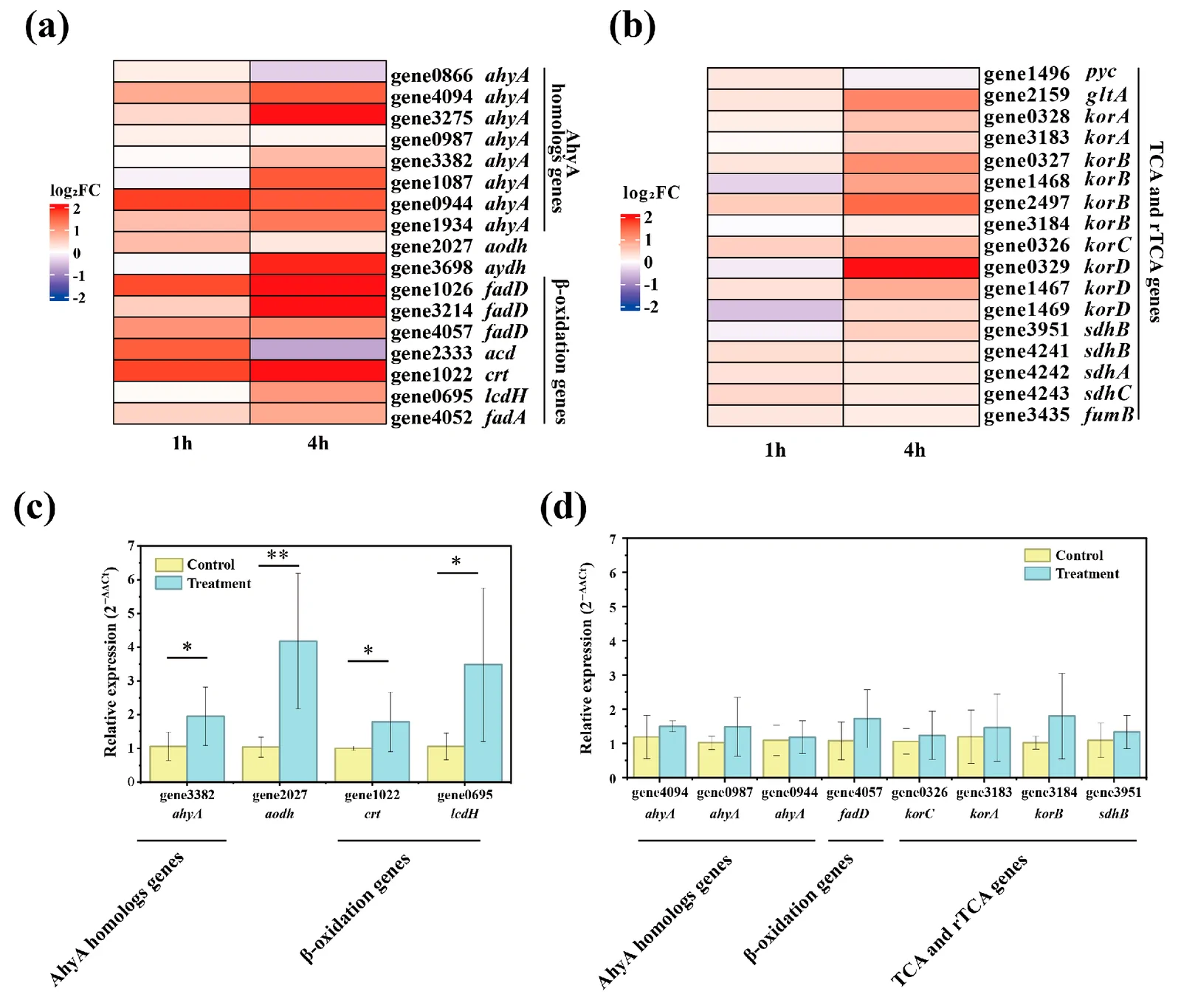
Comparative analysis of differentially expressed genes under hexadecane-induced and non-induced conditions at 1 h and 4 h. (**a**) A heatmap of differentially expressed genes involved in the initial oxidation of *n*-hexadecane and subsequent β-oxidation. Genes for initial oxidation include *ahyA* homologs, *aodh* (alcohol dehydrogenase), and *aydh* (aldehyde dehydrogenase). Genes for β-oxidation include *fadD* (long-chain fatty acid–CoA ligase), *acd* (acyl–CoA dehydrogenase family protein), *crt* (enoyl–CoA hydratase), *lcdH* (3-hydroxyacyl–CoA dehydrogenase), and *fadA* (acetyl–CoA acyltransferase). (**b**) A heatmap of differentially expressed genes involved in the tricarboxylic acid (TCA) and the reductive tricarboxylic acid (rTCA) cycles, including *pyc* (pyruvate carboxylase), *gltA* (citrate synthase), *kor* (2-oxoglutarate ferredoxin oxidoreductase or pyruvate: ferredoxin oxidoreductase), *sdh* (succinate dehydrogenase), and *fum* (fumarate hydratase). (**c**) RT-qPCR analysis of selected genes associated with AhyA homologs, alcohol dehydrogenase, and fatty acid β-oxidation. (**d**) RT-qPCR analysis of selected genes associated with AhyA homologs, β-oxidation, and central carbon metabolism. Data are presented as the mean ± standard deviation of three independent biological replicates. Asterisks indicate statistically significant differences between the additional *n*-hexadecane treatment and the control: *p* < 0.05 (*), *p* < 0.01 (**).

Transcriptomic analysis further revealed that several candidate genes identified in the genome were upregulated at 1 h and/or 4 h following *n*-hexadecane addition. Among the genes encoding AhyA homologs, genes 0944 and 4094 were upregulated at 1 h, with gene 0944 showing a log_2_FC of 1.8. At 4 h, genes 3275, 0944, 1087, 1934, and 3382 were upregulated (Figure 5a). Other oxidative enzymes coding genes were also upregulated: *aodh* (alcohol dehydrogenase) was upregulated at 1 h and 4 h, and *aydh* (aldehyde dehydrogenase) was upregulated at 4 h. Regarding β-oxidation, coding genes including *fadD* (long-chain fatty acid–CoA ligase), *acd* (acyl–CoA dehydrogenase), *crt* (enoyl–CoA hydratase), *lcdH* (3-hydroxyacyl–CoA dehydrogenase), and *fadA* (acetyl–CoA acyltransferase) were all upregulated at both 1 h and 4 h, with generally higher fold changes at 4 h (Figure 5a). For central carbon metabolism, genes involved in the TCA and rTCA cycles were also upregulated (Figure 5b). For instance, *kor* (2-oxoglutarate ferredoxin oxidoreductase or pyruvate: ferredoxin oxidoreductase), *sdh* (succinate dehydrogenase), and *fum* (fumarate hydratase) were upregulated at both 1 h and 4 h.

RT-qPCR analysis at 24 h showed significant upregulation of gene 3382, which encodes an AhyA-homologous protein, as well as *aodh* (2027), *crt* (1022), and *lcdH* (0695), following the additional *n*-hexadecane amendment (Figure 5c). Several other selected genes showed higher mean expression levels than those in the non-amended control, although the differences were not statistically significant (Figure 5d).

### 3.6. Metabolomic Responses and Assimilation of n-Hexadecane-Derived Carbon

Comparative metabolomic analysis was performed to determine whether the transcriptional responses were accompanied by corresponding metabolites changes (Supplementary Dataset). Hexadecanal was detected and exhibited a positive log_2_ fold change following *n*-hexadecane exposure (log_2_FC = 0.176). Among the detected fatty acids, tetradecanoic acid and dodecanoic acid also exhibited positive log_2_FC values of 0.228 and 0.413, respectively, whereas hexadecanoic acid exhibited a negative log_2_FC value of −0.371. The coordinated directional changes in the C16 aldehyde and C16-, C14-, and C12-fatty acids provide metabolite level observations consistent with fatty acid oxidation and chain-shortening reactions.

Stable-isotope labeling analysis was further performed to trace the intracellular fate of *n*-hexadecane-derived carbon. Following incubation with hexadecane-1,2-^13^C_2_, ^13^C incorporation was detected in fatty acid and lipid compounds, as well as in amino acid metabolites. Representative metabolites exhibiting relatively high ^13^C labeling are summarized in Table 1, including 9,10-dihydroxy-8-oxo-12-octadecenoic acid, N-palmitoyl-leucine, and N-Stearoyl Glutamic acid. Additional ^13^C-labeled lipid and amino acid metabolites are also listed in Table 1.

Overall, the detection of *n*-hexadecane-derived ^13^C in diverse intracellular metabolites demonstrates that carbon derived from *n*-hexadecane was incorporated into cellular constituents. When considered together with the comparative metabolomic and transcriptomic results, these findings support the involvement of fatty acid degradation-related metabolism in the response of strain L01 to *n*-hexadecane.

### 3.7. Nitrate Reduction During Microoxic Growth of Strain L01

To investigate potential electron acceptor utilization of strain L01 growing under the near-microoxic conditions, nitrate and sulfate concentrations were quantified during cultivation in BM1 supplemented with *n*-hexadecane at a final concentration of 0.05% (*v*/*v*). In inoculated cultures, nitrate concentration decreased from 20.24 ± 0.50 mM at 0 d to 12.83 ± 0.73 mM at 10 d, whereas nitrate concentration remained nearly unchanged in the uninoculated control, increasing from 19.96 ± 0.13 mM to 20.63 ± 0.08 mM over the same period (Table 2). In contrast, sulfate concentration showed only minor changes during incubation, increasing from 0.69 ± 0.01 mM to 0.90 ± 0.02 mM in inoculated cultures and remaining largely stable in the uninoculated control (Table 2). Consistent with the decrease in nitrate concentration, transcriptomic analysis showed that genes associated with nitrate reduction, including *napA*, *nirK*, *norB*, and *nrfH*, were upregulated following *n*-hexadecane addition (Supplementary Dataset). After correction for changes in the abiotic control, the net decrease in nitrate concentrations after 10 days was 8.08 mmol L^−1^.

## 4. Discussion

Physiological experiments showed that *n*-hexadecane promoted the growth of strain L01 under the near-microoxic conditions used in this study. In BM1, cultures supplemented with *n*-hexadecane exhibited faster growth than those without *n*-hexadecane. In a separate degradation assay, the residual amount of *n*-hexadecane decreased during cultivation, with a degradation rate of 10.4% after 10 d. Together, these observations support the ability of strain L01 to consume and assimilate *n*-hexadecane under near-microoxic conditions, although they do not demonstrate complete mineralization of the substrate.

Polyphasic taxonomic analyses further support the placement of strain L01 in a distinct genus-level lineage within the family *Symbiobacteriaceae*. This family currently comprises only a limited number of cultivated representatives isolated from environments such as compost, shellfish, and geothermal habitats [15,16,18]. To date, hydrocarbon utilization has not been reported for these cultivated representatives [16,17,18]. The isolation and laboratory characterization of strain L01 therefore extend the range of source environments and physiological traits documented for cultivated members of *Symbiobacteriaceae*. However, because the environmental abundance, distribution, and in situ activity of strain L01 or closely related microorganisms were not examined, their contribution to hydrocarbon transformation in hydrothermal sediments remains unknown.

To place the degradation performance of strain L01 in context, it is useful to compare it with previously reported alkane-degrading microorganisms. Under aerobic conditions, *n*-hexadecane degradation can occur relatively rapidly. For example, *Pseudomonas aeruginosa* PSA5, *Rhodococcus* sp. NJ2, and *Ochrobactrum intermedium* P2 removed 99%, 95%, and 92% of supplied *n*-hexadecane, respectively, after 10 d of incubation [38]. In contrast, anaerobic alkane degradation generally proceeds over longer timescales. The sulfate-reducing strain AK-01 degraded approximately 91% of *n*-hexadecane after 44 d, whereas strain Hxd3 required 330 d to achieve 89% degradation [39,40]. Similarly, a denitrifying enrichment culture consumed approximately 43 μmol of *n*-hexadecane in 50 mL medium after 52 d of incubation [41]. In the present study, strain L01 removed approximately 10.4%, corresponding to 2.17 μmol in a 10 mL culture, of the supplied *n*-hexadecane within 10 d of incubation. Although this removal was substantially lower than that reported for aerobic alkane degraders, the detectable consumption within 10 d indicates early-stage activity under near-microoxic conditions. Direct quantitative comparisons among studies should nevertheless be treated cautiously because substrate concentrations, inoculum sizes, media compositions, incubation temperatures, and analytical procedures differed considerably. The relatively short incubation period used here was also insufficient to determine the maximum degradation capacity of strain L01. Longer cultivation experiments under more strictly controlled conditions will therefore be required to characterize its degradation kinetics and final degradation extent.

Comparison with representative cultured bacteria capable of *n*-alkane degradation under anoxic conditions further illustrates the distinctive characteristics of strain L01 (Table S6). The nitrate-reducing strain HxN1 and the sulfate-reducing strain AK-01 activate *n*-alkanes through addition to fumarate, as supported by the detection of alkylsuccinate products and the identification of mas or ass genes [42,43]. In contrast, the nitrate-reducing strain HdN1 lacks recognizable mas/ass-like genes, and the proposed involvement of nitrate-derived oxidants and candidate monooxygenases in alkane activation remains hypothetical [44]. An EBDH-like hydroxylation model has also been proposed for the sulfate-reducing strain *Desulfococcus oleovorans* Hxd3, although its initial activation reaction remains unresolved [44,45]. The thermophilic sulfate reducer *Desulfothermus naphthae* TD3^T^ is more similar to strain L01 with respect to its growth temperature and hydrothermal origin, but it couples alkane oxidation to sulfate reduction rather than nitrate reduction, and its initial alkane activation mechanism has not been established [44]. In comparison with these organisms, strain L01 belongs to a phylogenetically distinct lineage within *Symbiobacteriaceae*, grows optimally at approximately 55 °C, and is capable of *n*-hexadecane utilization under near-microoxic, nitrate-reducing conditions. Its genome lacks the canonical genes associated with currently recognized anaerobic alkane activation mechanisms, including *assA*, *bssA*, *Acr*, and *Mcr*-like genes [46,47,48,49]. Because strain L01 also showed tolerance to low oxygen concentrations, it is more appropriately described as capable of alkane utilization under near-mirooxic conditions rather than as a strictly anaerobic bacterium. Its low-oxygen tolerance does not, however, establish that molecular oxygen participates in the initial activation of *n*-hexadecane.

The combined genomic, transcriptomic, and metabolomic analyses provide further insight into hydrocarbon-associated metabolism in strain L01. Although canonical genes associated with established anaerobic alkane activation pathways were not detected [46,47,48,49], eight genes encoding AhyA-homologous proteins were identified. AhyABC has recently been proposed to mediate oxygen-independent alkane hydroxylation through C2-methylene activation [49,50]. AhyA homologs were transcriptionally upregulated following *n*-hexadecane addition at both early time points. Genes encoding alcohol dehydrogenase, aldehyde dehydrogenase, and multiple enzymes involved in β-oxidation were also upregulated. In addition, metabolomic analysis detected changes in hexadecanal and several fatty acid-related metabolites. Collectively, these responses are consistent with an alkane-associated metabolic response involving alcohol and aldehyde oxidation followed by fatty acid degradation.

However, sequence similarity, transcriptional upregulation, and metabolite changes do not establish the catalytic activity or substrate specificity of the candidate proteins. The identified AhyA homologs should therefore be regarded as candidate AhyA-like proteins rather than confirmed alkane hydroxylases. Likewise, the available results do not establish a complete AhyA-mediated degradation pathway. Direct verification would require heterologous expression and purification of the candidate proteins, in vitro activity assays with *n*-hexadecane, identification of reaction products, and ideally functional analyses of the predicted catalytic residues [51].

The ^13^C labeling experiments demonstrated the incorporation of *n*-hexadecane-derived carbon into fatty acids, membrane lipids, and amino acid-related metabolites. These observations provide direct evidence that carbon derived from *n*-hexadecane entered intracellular metabolic pools. Nevertheless, isotopic incorporation into biomass does not, by itself, demonstrate that strain L01 conserved energy directly from *n*-hexadecane oxidation. Carbon assimilation and energy conservation represent related but distinct physiological processes. Confirmation of the energetic coupling would require complementary measurements such as measurements of substrate-dependent ATP production, respiratory activity, electron transport activity, or growth yields obtained in a defined cultivation system.

A complete carbon mass balance was not established in the present study. The incubation system was not designed to quantitatively recover carbon from the headspace, dissolved inorganic carbon, extracellular metabolites, residual substrate, and total biomass. Consequently, the proportion of consumed *n*-hexadecane that was incorporated into biomass, converted into soluble metabolites, or mineralized to CO_2_ remains unknown. The results therefore support biological consumption and intracellular assimilation of *n*-hexadecane-derived carbon but should not be interpreted as evidence of complete mineralization. Future experiments combining ^13^C-labeled *n*-hexadecane with quantitative measurements of residual substrate, ^13^CO_2_, dissolved inorganic carbon, soluble metabolites, and biomass carbon will be necessary to establish a complete carbon balance.

Nitrate introduced from surrounding seawater is considered an important terminal electron acceptor supporting anaerobic microbial metabolism in hydrothermal environments [52,53]. Consistent with this environmental context, nitrate reduction appeared to be an important component of the physiology of strain L01. During cultivation, nitrate concentration decreased substantially, whereas sulfate remained largely unchanged. In addition, genes involved in nitrate reduction, including *napA*, *nirK*, *norB*, and *nrfH*, were transcriptionally upregulated following *n*-hexadecane addition. These observations indicate that nitrate reduction occurred during cultivation and may have contributed to metabolism of strain L01.

A theoretical electron balance comparison was performed using the background-corrected changes measured after 10 days. The net *n*-hexadecane depletion was 0.2169 mmol L−1, whereas the net nitrate depletion was 8.08 mmol L^−1^, corresponding to an apparent ratio of approximately 37.3 mol nitrate per mol *n*-hexadecane. According to the stoichiometric equation reported by Ehrenreich et al. [54], complete oxidation of *n*-hexadecane to CO_2_ coupled to nitrate reduction to N_2_ theoretically requires 19.6 mol nitrate per mol *n*-hexadecane. Thus, the measured *n*-hexadecane depletion would theoretically account for approximately 4.25 mmol L^−1^ nitrate reduction, which is lower than the observed nitrate depletion. This discrepancy is most likely explained, at least in part, by the composition of BM1. Yeast extract, tryptone, and sodium acetate could provide additional electron donors for nitrate reduction. Although the abiotic control corrected for non-biological changes, it could not distinguish nitrate reduction supported by these auxiliary organic substrates from nitrate reduction coupled specifically to *n*-hexadecane oxidation. Consequently, a direct stoichiometric coupling between *n*-hexadecane oxidation and nitrate reduction could not be established under the tested conditions. This limitation is also consistent with the slow growth observed when *n*-hexadecane was supplied as the sole major carbon source in the defined MSM1 medium (Figure 4a). Future experiments using defined media, longer incubation periods, and quantitative measurements of nitrogenous products will be required to determine the stoichiometric relationship between *n*-hexadecane oxidation and nitrate reduction.

Overall, this study identifies strain L01 as a cultivated member of *Symbiobacteriaceae* capable of consuming and assimilating *n*-hexadecane-derived carbon under near-mirooxic conditions. The physiological and multi-omics results expand the known metabolic diversity of cultivated members of this family and identify several candidate proteins and downstream metabolic responses potentially associated with *n*-hexadecane utilization. However, the initial activation enzyme, the complete degradation pathway, the energetic coupling to growth, and the stoichiometric relationship with nitrate reduction remain unknown. Strain L01 therefore provides a useful cultivated model for further investigation of thermophilic alkane metabolism in the family *Symbiobacteriaceae*.

## 5. Provisional Taxonomic Descriptions

### 5.1. Provisional Description of “Thermalkanevorax”

“*Thermalkanevorax*” (Ther.mal.ka.ne.vo’rax. Gr. fem. n. thermē, heat; N.L. neut. n. alkanum, alkane; L. masc. adj. vorax, devouring; N.L. masc. n. *“Thermalkanevorax*”, a heat-loving, alkane-devouring bacterium, referring to its thermophilic lifestyle and ability to utilize *n*-hexadecane).

Cells are Gram stain-positive (Figure S2), rod-shaped, and grow under nearly microoxic conditions. Members of the genus are thermophilic, can grow at neutral pH. *n*-Hexadecane can be utilized as a carbon source.

Phylogenetically, the proposed genus *“Thermalkanevorax*” forms a distinct lineage within the family *Symbiobacteriaceae* clearly separated from all currently described genera based on 16S rRNA gene phylogeny and average amino acid identity (AAI) analyses.

### 5.2. Provisional Description of “Thermalkanevorax longiformis”

“*Thermalkanevorax longiformis*” (lon.gi.for’mis. L. adj. *longiformis*, long-shaped, referring to the elongated cell morphology).

Shows the following characteristics in addition to those given for the genus: Cells are Gram stain-positive rods (Figure S2) measuring approximately 10–20 μm in length and ~0.2 μm in diameter. Growth occurs under nearly microoxic conditions at 55 °C and at pH 7.0. *n*-Hexadecane can be utilized as a carbon source.

The genomic DNA G+C content of strain L01 is 65.75 mol%.

Strain L01 (MCCC 1K10379) was isolated from a hydrothermal sediment sample collected from the Mid-Atlantic Ridge.

## 6. Conclusions

In this study, a novel thermophilic bacterium, provisionally named *“Thermalkanevorax longiformis*” (strain L01), was isolated from a hydrothermal vent along the Mid-Atlantic Ridge. Strain L01 represents the first cultivated member of the family *Symbiobacteriaceae* shown to degrade *n*-hexadecane under nearly microoxic conditions. Integrated physiological, transcriptomic, and metabolomic analyses demonstrated active hydrocarbon degradation, carbon assimilation, and metabolic responses to *n*-hexadecane. Collectively, these findings expand the known metabolic repertoire of *Symbiobacteriaceae* and establish strain L01 as a valuable model for investigating hydrocarbon metabolism in thermophilic bacteria.

Accordingly, the present study characterizes the physiological and molecular responses of strain L01 under laboratory cultivation conditions; however, its environmental abundance, in situ activity, and ecological relevance remain to be determined.

## Figures and Tables

**Table 1:** Representative intracellular metabolites exhibiting relatively high ^13^C labeling following cultivation of strain L01 with hexadecane-1,2-^13^C_2_ as a tracer.

Category	Metabolite	Labeling Ratio (%)
Fatty acids metabolism	9,10-Dihydroxy-8-oxo-12-octadecenoic acid	64.3 ± 28.1
	N-palmitoyl-leucine	41.9 ± 28.5
	N-Stearoyl Glutamic acid	34.9 ± 13.7
Lipids	phosphatidylethanolamine (1-cis-vaccenoyl, 2-palmitoleoyl)	100 ± 0
	1-pentadecanoyl-2-(11Z-eicosenoyl)-glycero-3-phosphocholine	76.8 ± 12.3
	1-Octadecanoyl-2-(5Z,8Z,11Z,14Z-eicosatetraenoyl)-sn-glycero-3-phosphoethanolamine	76.7 ± 26.09
	3-Dehydrosphinganine	58.1 ± 22.0
	PC(20:3(8Z,11Z,14Z)/14:1(9Z))	55.4 ± 37.3
	1-Hexadecanoyl-2-(9Z-octadecenoyl)-sn-glycero-3-phosphoethanolamine	52.1 ± 31.1
	PC(35:1)	51.3 ± 38.3
	PC(18:1(9Z)/14:0)	46.8 ± 28.3
	Hexadecasphinganine	42.7 ± 46.7
	PC(14:0/14:0)	42.4 ± 31.9
	N-(2-hydroxyhexadecanoyl)-phytosphingosine	41.1 ± 15.1
Amino acid metabolism	N-Linoleoyl Isoleucine	32.4 ± 15.2
	3-Phenylpropanoic acid	7.76 ± 5.0

**Table 2:** Changes in nitrate and sulfate concentrations and their net consumption during cultivation.

Culture	Initial Concn (mM)	Concn (mM) at 10 d	Net Consumption (%)
NO_3_^−^ (Inoculated)	20.24 ± 0.50	12.83 ± 0.73	36.6
NO_3_^−^ (Uninoculated)	19.96 ± 0.13	20.63 ± 0.08	−3.4
SO_4_^2−^ (Inoculated)	0.69 ± 0.01	0.90 ± 0.02	−30.4
SO_4_^2−^ (Uninoculated)	0.65 ± 0.04	0.67 ± 0.01	−3.1

## Data Availability

The 16S rRNA and genome sequences of strain L01 have been deposited in the NCBI database under accession numbers PX804844 and PRJNA1347602. The raw transcriptomic sequencing data have been deposited in the NCBI Sequence Read Archive under the same BioProject accession number (PRJNA1347602).

## Supplementary Materials

The following supporting information can be downloaded at https://www.mdpi.com/article/10.3390/microorganisms14081783/s1: Figure S1: Transmission electron micrograph of strain L01; Figure S2: Gram staining of strain L01 observed by light microscopy; Figure S3: Standard curve for quantitative PCR based on 16S rRNA gene (from the strain L01) copy number; Figure S4: Standard curve of *n*-hexadecane; Figure S5: Circular genome map of strain L01 with a total length of 4.3 Mbp; Figure S6: Conserved motifs in AhyA-homologous proteins; Table S1: Modified Wolin’s mineral solution; Table S2: Digital DNA–DNA hybridization (dDDH) values; Table S3: Characteristics between strain L01 and type strains of the family *Symbiobacteriaceae*; Table S4: Concentrations of individual *n*-alkanes detected in the hydrothermal sediment from which strain L01 was isolated; Table S5: Putative genes encoding anaerobic alkane C2-methylene hydroxylase (AhyA) identified in the genome of strain L01; Table S6: Comparison of strain L01 with representative cultured bacteria capable of *n*-alkane degradation under anoxic conditions; Table S7: primer for RT-qPCR. Workbook S1: Supplementary Dataset. References [55,56] are cited in the supplementary materials.

## Abbreviations

The following abbreviations are used in this manuscript:

AAI	Average amino acid identity
ANI	Average nucleotide identity
dDDH	Digital DNA-DNA hybridization
BM	Basal medium
BM1	Basal medium 1
TEM	Transmission electron microscopy
SEM	Scanning electron microscopy
qPCR	Quantitative real-time PCR
GC-MS	Gas chromatography–mass spectrometry
LC-MS/MS	Liquid chromatography–tandem mass spectrometry
