# Supplementary material for: A Novel n-Hexadecane-Degrading Bacterium Provisionally Named “Thermalkanevorax longiformis” Isolated from a Deep-Sea Hydrothermal Vent
Source: Microorganisms. 2026 Aug 13;14(8):1783. doi: 10.3390/microorganisms14081783 (PMC13515206; doi:10.3390/microorganisms14081783)
Supplement: Supplementary file 1 [file microorganisms-14-01783-s001.zip › Supplementary Material.pdf]

A Novel *n*-Hexadecane-Degrading Bacterium provisionally Named "*Thermalkanevorax longiformis*" Isolated from a Deep-Sea Hydrothermal Vent

Huilin Wen[1, 2, 3], Ge Liu[1, 2], Chaomin Sun*[1, 2], Rui Liu*[1, 2]

[1]Laboratory of Experimental Marine Biology & Center of Deep Sea Research, Shandong Province Key Laboratory of Marine Biodiversity and Bio-resource Sustainable Utilization, Institute of Oceanology, Chinese Academy of Sciences, Qingdao 266071, China

[2]Laboratory for Marine Biology and Biotechnology, Qingdao National Laboratory for Marine Science and Technology, Qingdao 266071, China

[3]College of Earth Science, University of Chinese Academy of Sciences, Beijing 100049, China

* Correspondence:

Chaomin Sun Tel.: +86 532 82898857; fax: +86 532 82898857

E-mail address: sunchaomin@qdio.ac.cn

Rui Liu Tel.: +86 532 82898648; fax: +86 532 82898857

E-mail address: liurui@qdio.ac.cn

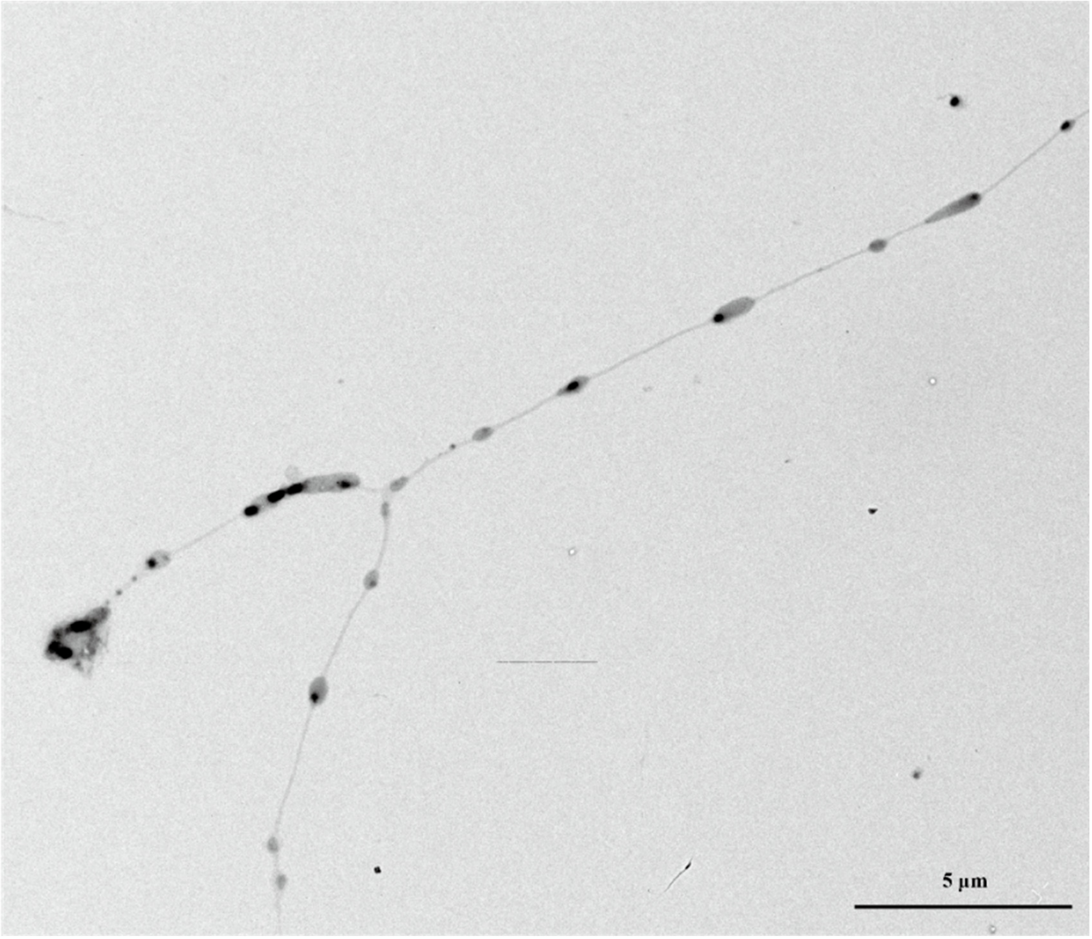

Figure S1. Transmission electron micrograph of strain L01. After entering the stationary growth phase, strain L01 formed necklace-like filamentous chains composed of serially aligned spherical bodies.

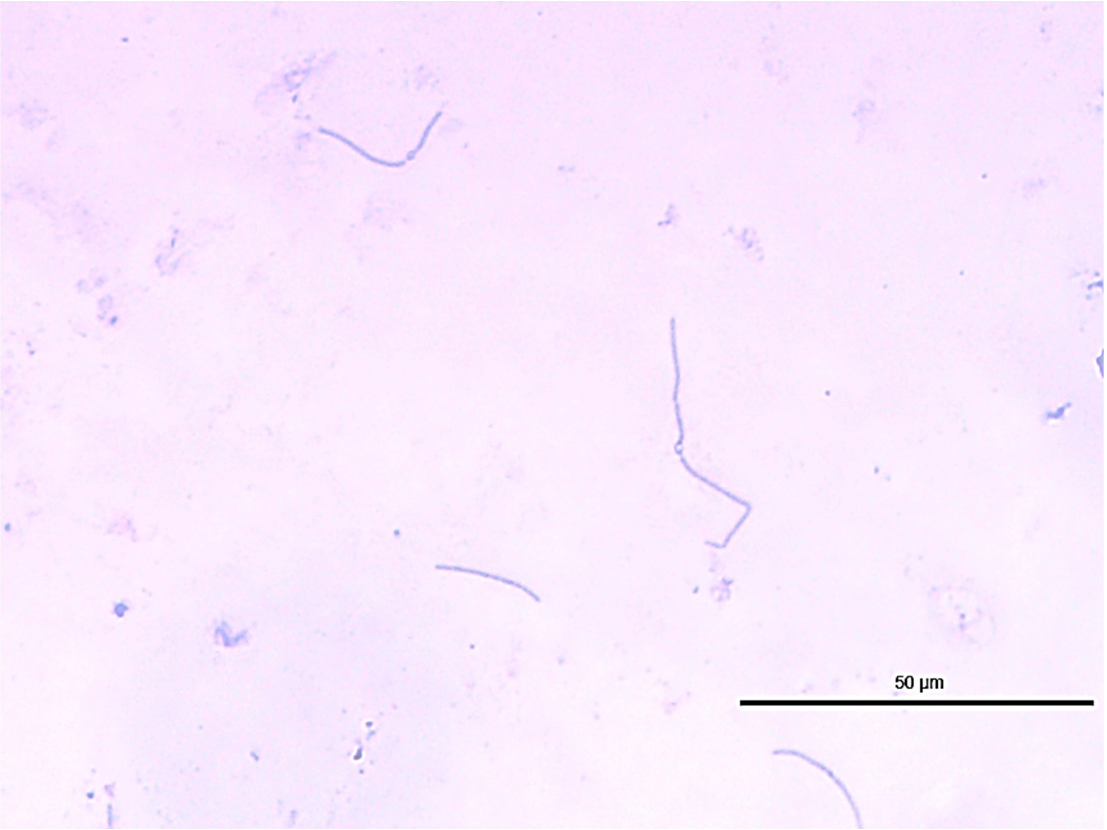

Figure S2. Gram staining of strain L01 observed by light microscopy. Cells exhibited a Gram-positive reaction and were visualized using a 100 × oil-immersion objective.

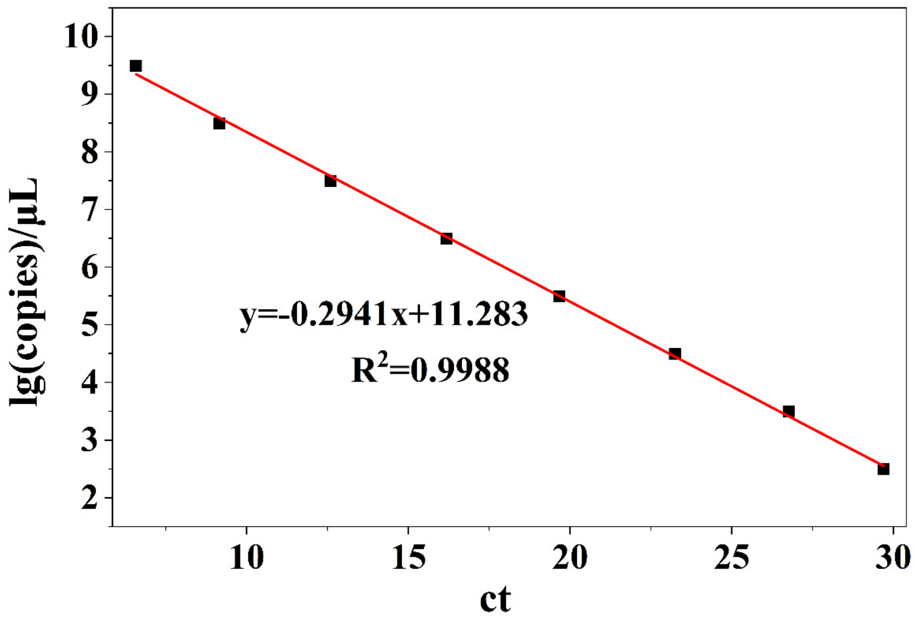

Figure S3. Standard curve for quantitative PCR based on 16S rRNA gene (from the strain L01) copy number. The standard curve was generated by plotting Ct values against the recombinnant plasmid copy numbers.

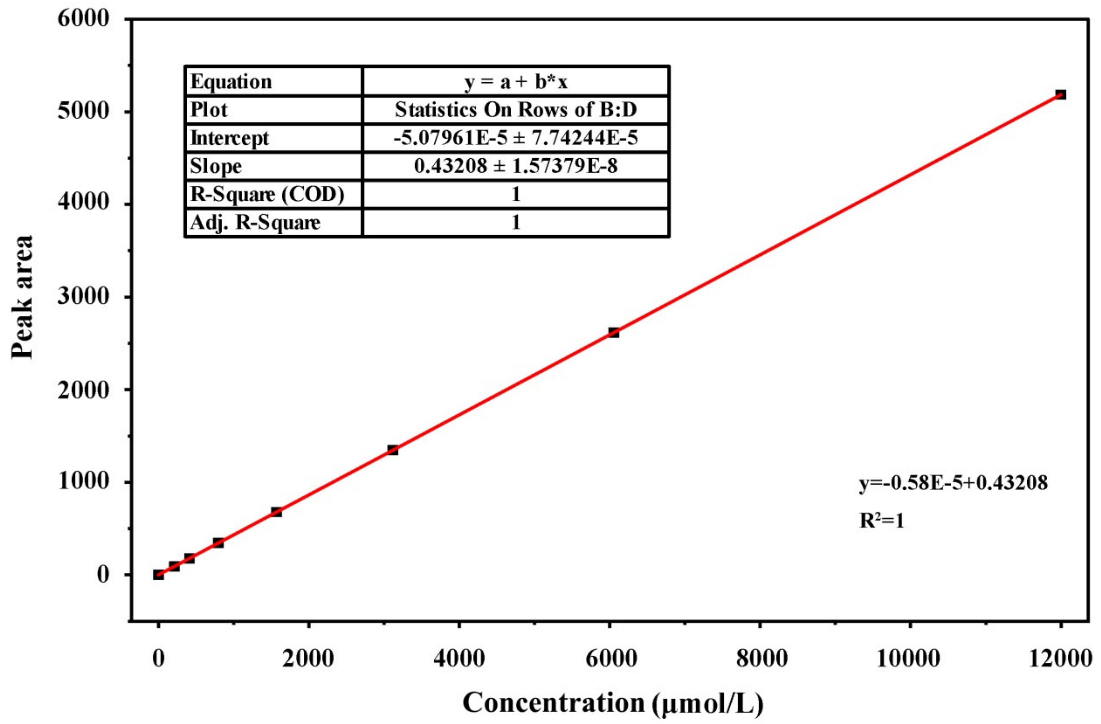

Figure S4. Standard curve of *n*-hexadecane. A standard curve was generated by analyzing serially diluted *n*-hexadecane standards (0-11,997.77 μmol/L), and linear regression was performed using the measured peak areas versus concentrations.

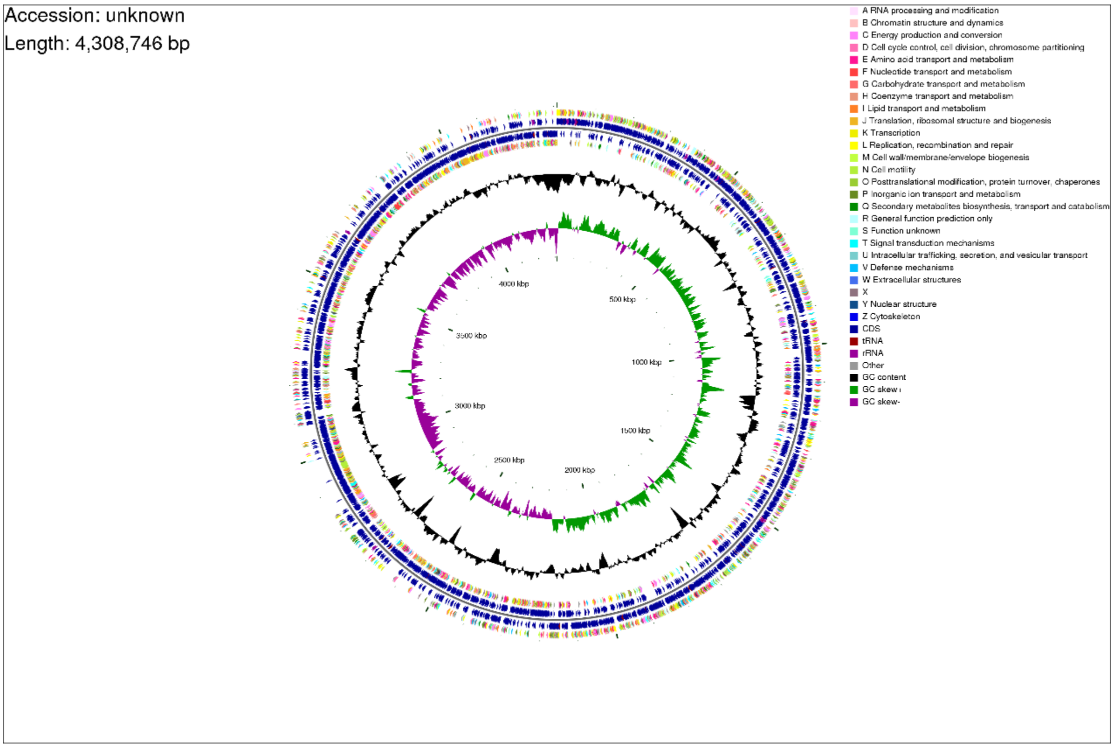

Figure S5. Circular genome map of strain L01 with a total length of 4.3 Mbp. The outermost circle represents annotated genes, color-coded by functional category according to the COG (Clusters of Orthologous Groups) classification system. Inner circles depict GC content (black), GC skew (green: positive; purple: negative), and tRNA/rRNA genes (brown and purple, respectively). The scale indicates genomic positions in kilobases.

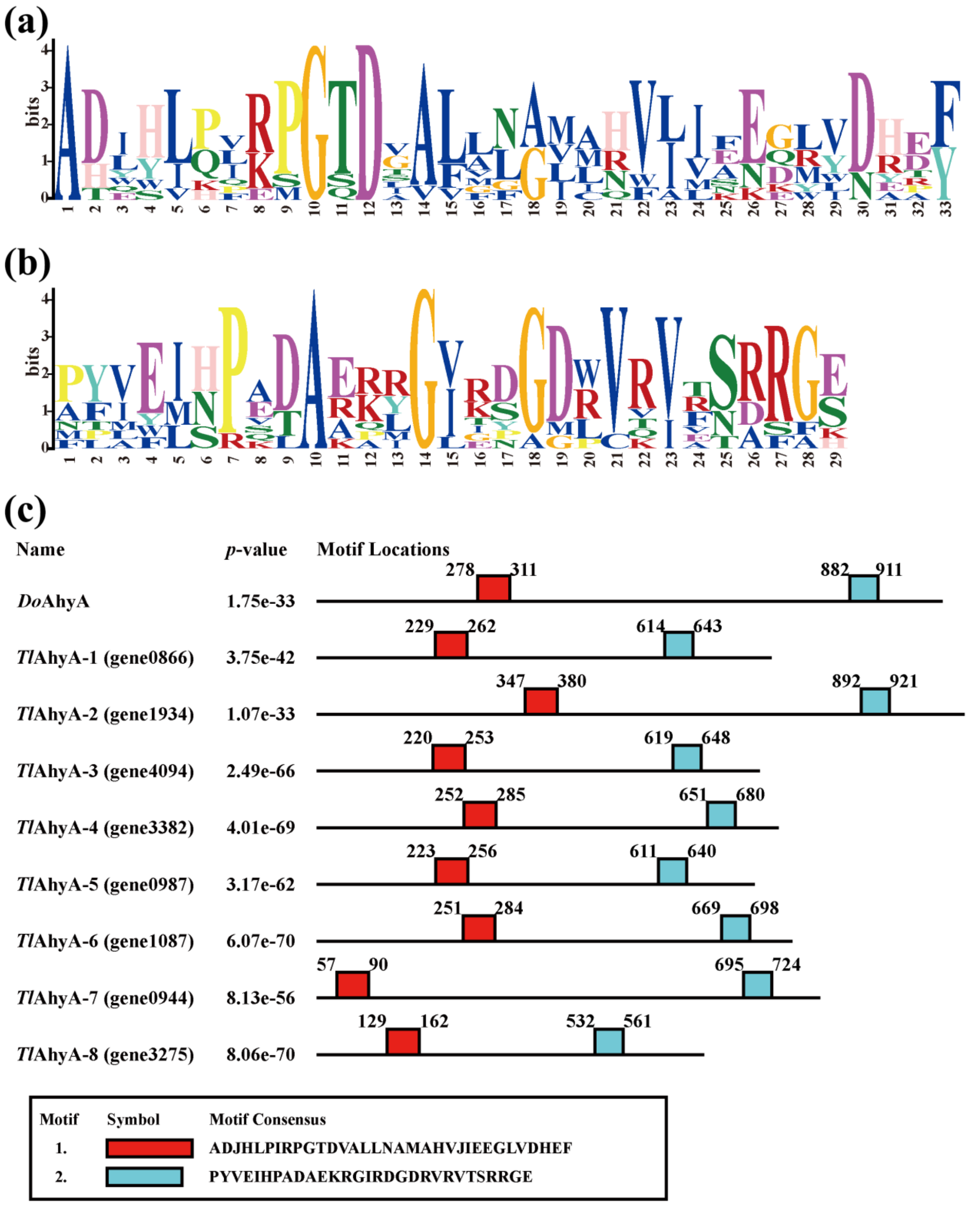

Figure S6. Conserved motifs in AhyA homologous proteins. (A, B) Two conserved motifs discovered by the MEME Suite. (C) The location of each motif in every AhyA homologous proteins.

Table S1 Modified Wolin's mineral solution.

Compound	Concentration (g/L)	Concentration (mM)
Nitrilotriacetic acid	1.50	7.848
$MgSO_4·7H_2O$	3.00	12.172
$MnSO_4·H_2O$	0.50	2.958
NaCl	1.00	17.112
$FeSO_4·7H_2O$	0.10	0.36
$CoSO_4·7H_2O$	0.18	1.161
$CaCl_2$	0.08	0.68
$ZnSO_4·7H_2O$	0.18	0.626
$CuSO_4·5H_2O$	0.01	0.04
$AlK(SO_4)_2·12H_2O$	0.02	0.042
H_3BO_3	0.01	0.162
$Na_2MoO_4·2H_2O$	0.01	0.041
$NiCl_2·6H_2O$	0.03	0.231
Na_2SeO_3	0.0002	0.001
$Na_2WO_4·2H_2O$	0.0004	0.001

First dissolve nitrilotriacetic acid to 1000 mL distilled water and adjust pH to 6.5 with KOH, then add minerals. Adjust final to pH 7.0 with KOH.

Table S2 Digital DNA–DNA hybridization (dDDH) values. The dDDH values were estimated using the Genome-to-Genome Distance Calculator (GGDC). Results are given for Formula 1, Formula 2, and Formula 3.

Query genome	Reference genome	dDDH (Formula 1, %)	dDDH (Formula 2, %)	dDDH (Formula 3, %)
L01_Chromosome	GCA_000009905.1	14.8	20.2	14.9
L01_Chromosome	GCA_003242675.2	13.5	19.3	13.8
L01_Chromosome	GCF_017874315.1	15.1	20.2	15.2
L01_Chromosome	GCF_019806665.1	14.3	20.1	14.5
L01_Chromosome	GCF_040797425.1	14.4	19.5	14.6
L01_Chromosome	AP042433.1	14.6	20.8	14.8
L01_Chromosome	AP042454.1	14.8	20.5	15
L01_Chromosome	GCF_000183545.2	12.8	20.5	13.2
L01_Chromosome	GCF_000184705.1	12.8	20.1	13.2
L01_Chromosome	GCF_004634385.1	12.7	20.3	13.1
L01_Chromosome	GCA_036564035.1	12.5	17	12.9
L01_Chromosome	GCF_033582035.1	12.7	20.8	13.1
L01_Chromosome	GCF_025999835.1	13	20.5	13.4

Table S3 Characteristics between strain L01 and type strains of the family *Symbiobacteriaceae*.

Characteristic	L01	*Symbiobacterium turbinis* JCM 15996	*S. thermophilum* IAM 14863	*S. ostreiconchae* JCM 15048	*S. terraclitae* JCM 15997	*Caldinitratiruptor microaerophilus* JCM 16183
Isolation source	Hydrothermal vent	Horned turban shell	Compost	Oyster shell	Barnacle	Hot spring
Gram staining	Gram positive	Gram negative	Gram negative	Gram negative	Gram negative	Gram negative
Cell length (μm)	10.0-20.0	5.2-14.5	1.7-7.0	3.3-7.1	2.9-11.7	2.0-3.0
Cell width (μm)	0.2	0.3-0.4	0.3-0.4	0.3-0.4	0.3-0.4	0.2
Motility	+	-	-	-	+	-
Endospore-like structure formation	+	-	-	+	+	-
Optimum temperature	55 °C	60 °C	60 °C	55 °C	55 °C	65 °C
O_2 concentration for growth (% v/v)	41 (μmol/L)	0	0-2.0	0	0	2.0
NaCl concentration for growth (% w/v)	1-2.5	0-4.0	0-2.0	0-1.0	0-2.0	0-1.0
G+C content (mol%)	65.75	67.2	65.1	68.8	67.1	70.3
Electron acceptor	Nitrate	Nitrate	ND	Nitrate	Nitrate	Nitrate
Growth on						
n-Hexadecane	+	ND	ND	ND	ND	ND

+, Positive; -, negative; W, weakly positive; ND, no data available.

Table S4 Concentrations of individual *n*-alkanes detected in the hydrothermal sediment from which strain L01 was isolated.

***n*-Alkane**	**Concentration (ng/g)**
n-C9	77.4
n-C10	340.6
n-C11	138.6
n-C12	57.2
n-C13	85.8
n-C14	35.5
n-C15	51.9
n-C16	449.8
n-C17	49.4
n-C18	178.3
n-C19	47.3
n-C20	509.5
n-C21	72.6
n-C22	167.3
n-C23	179.8
n-C24	240.6
n-C25	329.8
n-C26	273.3
n-C27	365.6
n-C28	260.7
n-C29	312.9
n-C30	73.2
n-C31	99.5
n-C32	7.2
**Total (C9-C32)**	**4403.7**

Table S5 Putative genes encoding anaerobic alkane C2-methylene hydroxylase (AhyA) identified in the genome of strain L01.

Gene ID	Query name	E-value	Score
gene0866	AhyA	1.00E-59	200.05
gene1934	AhyA	3.5E-32	109.3
gene4094	AhyA	4.8E-27	92.3
gene3382	AhyA	1.6E-26	90.5
gene0987	AhyA	2.1E-26	90.2
gene1087	AhyA	1.1E-21	74.6
gene0944	AhyA	4.00E-18	62.8
gene3275	AhyA	2.40E-15	53.6

Table S6 Comparison of strain L01 with representative cultured bacteria capable of *n*-alkane degradation under anoxic conditions.

Strain	Temperature and condition	Electron acceptor	Alkane range	Supported activation-related evidence	Reference
L01	Thermophilic; optimum approximately 55°C; under near-microoxic conditions; growth also observed at anaerobic conditions.	Nitrate	*n*-Hexadecane examined	Canonical anaerobic activation genes not detected; AhyA-homologs proteins identified, but their function remains unverified	This study
HxN1	Cultivated at 28°C under strictly anoxic conditions	Nitrate	C_6–C_8	Fumarate addition; methylalkylsuccinate detected; *masABCDEFG* identified	(1)(2)
HdN1	Anoxic nitrate- or nitrite-reducing growth;	Nitrate or nitrite	C_6–C_{30}	*mas/ass* absent; candidate monooxygenases present; nitrate-derived oxidant or transient O_2 involvement hypothesized but unproven	(3)
AK-01	Mesophilic; optimum 26–28°C	Sulfate	C_{13}–C_{18}	Subterminal fumarate addition; two *ass* loci identified	(4)(5)
Hxd3	Mesophilic; optimum approximately 30°C	Sulfate	C_{12}–C_{20}	EBDH-like hydroxylation models remain unresolved	(4)(6)
TD3^T	Thermophilic; optimum approximately 60°C	Sulfate	C_6–C_{16}	Initial activation mechanism unresolved	(4)

1. Rabus, R.; Wilkes, H.; Behrends, A.; Armstroff, A.; Fischer, T.; Pierik, A.J.; Widdel, F. Anaerobic initial reaction of *n*-alkanes in a denitrifying bacterium: Evidence for (1-methylpentyl)succinate as initial product and for involvement of an organic radical in *n*-hexane metabolism. *J. Bacteriol.* 2001, 183, 1707–1715.
2. Grundmann, O.; Behrends, A.; Rabus, R.; Amann, J.; Halder, T.; Heider, J.; Widdel, F. Genes encoding the candidate enzyme for anaerobic activation of n-alkanes in the denitrifying bacterium, strain HxN1. *Environ. Microbiol.* 2008, 10, 376–385.
3. Zedelius, J.; Rabus, R.; Grundmann, O.; Werner, I.; Brodkorb, D.; Schreiber, F.; Ehrenreich, P.; Behrends, A.; Wilkes, H.; Kube, M.; Reinhardt, R.; Widdel, F. Alkane degradation under anoxic conditions by a nitrate-reducing bacterium with possible involvement of the electron acceptor in substrate activation. *Environ. Microbiol. Rep.* 2011, 3, 125–135.

4. So, C.M.; Young, L.Y. Isolation and characterization of a sulfate-reducing bacterium that anaerobically degrades alkanes. *Appl*. *Environ*. *Microbiol*. 1999, 65, 2969–2976.
5. Callaghan, A.V.; Wawrik, B.; Ní Chadhain, S.M.; Young, L.Y.; Zylstra, G.J. Anaerobic alkane-degrading strain AK-01 contains two alkylsuccinate synthase genes. *Biochem. Biophys. Res. Commun.* 2008, 366, 142–148.
6. Callaghan, A.V. Enzymes involved in the anaerobic oxidation of *n*-alkanes: From methane to long-chain paraffins. *Front. Microbiol.* 2013, 4, 89.
7. Teske, A.; Callaghan, A.V.; LaRowe, D.E. Biosphere frontiers of subsurface life in the sedimented hydrothermal system of Guaymas Basin. *Front*. *Microbiol*. 2014, 5, 362.

Table S7 primer for RT-qPCR.

Gene ID	Primer name	Primer sequence(5'-3')
3382	3382F	GGATCGGGTATTCGTCGTCC
	3382R	CAGTACAAGGGGCTCCAGTG
2027	2027F	ATGAGTACGTCGGCATCGTG
	2027R	GCCCCCACTCTGACATTGAT
1022	1022F	AGGCGATCTACGTTGCGG
	1022R	GATACCCCCTCCATCGCATC
0695	0695F	GGGGTACGTGGTCAACCG
	0695R	CTCGTAGGTCAGAAGCGGC
4094	4094F	AAGATGTCGGTCCAGGGGTT
	4094R	ACCGAAAAGTGCTACTGGGC
0987	0987F	GGGTCCACCAGGATCAGTTT
	0987R	CATGTCCAACTCCATCGGGG
094	0944F	TCCTTCAGGATGTTGCCGTC
	0944R	TACCTGATCCACCCGGACTT
4057	4057F	GGAGATACAGGCGTCGATGG
	4057R	CAGACGCTGAAGCTGATCCA
0326	0326F	AACTCCTTGGCGATGTCAGT
	0326R	CATCGTGATGAACCAGCCCT
3183	3183F	CATCTGGTCGATCAGGCGAT
	3183R	GCGGCTTGATATCCTCGTCA
3184	3184F	TCAAGTCCGAGCTGGTCAAC
	3184R	CGGTCTTCGTAACGGGTCAT
3951	3951F	TTGTCACCCTTCCGCATCAC
	3951R	CAGTGCCACGAGACCTTTGA
